# Longer recreational screen time contributes to the risk of age-related macular degeneration: a UK Biobank cohort study and two-sample Mendelian randomisation

**DOI:** 10.7189/jogh.15.04029

**Published:** 2025-01-31

**Authors:** Yikeng Huang, Xinyu Zhang, Li Liang, Yujin Jiang, Bo Li, Xinyu Zhu, Chenxin Li, Chufeng Gu, Wenjun Zou, Zhi Zheng, Shuzhi Zhao

**Affiliations:** 1 Department of Ophthalmology, Shanghai General Hospital, Shanghai Jiao Tong University School of Medicine, Shanghai, China; 2 National Clinical Research Centre for Eye Diseases, Shanghai, China; 3 Shanghai Key Laboratory of Ocular Fundus Diseases, Shanghai, China; 4 Shanghai Engineering Centre for Visual Science and Photomedicine, Shanghai, China; 5 Shanghai Engineering Centre for Precise Diagnosis and Treatment of Eye Diseases, Shanghai, China; 6 Department of Ophthalmology, The Fourth Affiliated Hospital of Soochow University, Suzhou, China; 7 Wuxi No. 2 People’s Hospital, Jiangnan University Medical Centre, Wuxi, China; 8 Ningde Municipal Hospital of Ningde Normal University, Fujian Medical University, Ningde, China; 9 Fujian Medical University, Fuzhou, China

## Abstract

**Background:**

Recreational screen time (RST) has been found to be associated with cognitive decline and neurodegenerative diseases. However, the association between RST and age-related macular degeneration (AMD), an ocular neurodegenerative disease, is still unclear. We aimed to elucidate the association between RST and AMD.

**Methods:**

This study consisted of three parts: 1) a prospective cohort study with 482 939 UK Biobank participants and a 12.8-year median follow-up to explore the association between RST and incident AMD; 2) a two-sample Mendelian randomisation (MR) analysis using summary-level genome-wide association analysis data for RST based on 526 725 European individuals to assess causality between RST and AMD; and 3) a cross-sectional study involving 38 478 UK Biobank individuals with optical coherence tomography data to investigate the link between RST and retinal thickness.

**Results:**

Multivariable Cox proportional-hazards models showed that an increase in total daily RST was associated with a greater risk of developing AMD (hazard ratio (HR) per standard deviation (SD) increase = 1.05; 95% confidence interval (CI) = 1.03, 1.07, *P* < 0.001). With further analysis, we revealed that daily high RST (>4 h/d) significantly increased the risk of AMD compared with low RST (0–3 h/d) (HR = 1.09; 95% CI = 1.04, 1.15, *P* = 0.001), while moderate RST (3.5–4 h/d) had no significant effect on AMD. Restricted cubic spline plots revealed a linear dose-response association between RST and AMD. With MR analysis we confirmed the potential causal association between RST and AMD (odds ratio per SD = 1.26; 95% CI = 1.01, 1.59, *P* = 0.042). Multivariable linear models suggested that an increase in RST was associated with thinning of the outer and inner retinal layers and total macular thickness.

**Conclusions:**

Longer RST may be a potential causal risk factor for AMD and may lead to pathological retinal thinning. Reducing RST could be beneficial for preventing AMD, and future research should identify the most effective time points for intervening on RST.

Age-related macular degeneration (AMD) is the leading cause of severe vision loss in elderly individuals aged >55 years in developed countries [1]. Approximately 196 million individuals, or 8.69% of the global population, are affected by AMD. With the increase in life expectancy and population ageing, this number is expected to rise to 288 million by 2040, making AMD a significant public health issue [1]. AMD is known to be a complicated disease that is affected by both genetic and environmental factors. Despite numerous studies, there is still limited understanding of the controllable risk factors for AMD, with the only identified modifiable risk factor being smoking [2]. This lack of knowledge hinders the clinical prevention and treatment of this disease. Therefore, it is crucial to identify other modifiable risk factors to ensure effective management of AMD.

Electronic devices such as smartphones and computers have become indispensable parts of people’s daily routines. Even elderly individuals have embraced modern habits such as watching television and videos and browsing the internet, which has increased recreational screen time (RST). Unfortunately, studies have suggested that an increase in RST may negatively impact human health. Specifically, excessive RST may promote cognitive decline and brain structure ageing in elderly individuals, leading to neurodegenerative diseases such as dementia and Parkinson disease, although these studies are mostly limited by their cross-sectional nature and are susceptible to residual confounding and reverse causality [3–6]. As a representative ocular neurodegenerative disease, AMD has clinical and pathological features similar to those of Alzheimer disease [7]. However, the role of RST in AMD remains unclear, and there is a lack of high-quality evidence supporting the effect of RST on neurodegenerative diseases.

In this study, we aimed to investigate the association between RST and the development of AMD. To achieve this goal, we used a large AMD cohort and optical coherence tomography (OCT) data from the UK Biobank to explore the impact of RST on the onset of AMD and the possible changes in the thickness of retinal layers. Moreover, we investigated the varying effects of different durations of RST on AMD and the shape of the exposure-outcome relationship. Further, we employed Mendelian randomisation (MR) analysis, which uses genetic variants as instrumental variables and is thought to overcome confounding and reverse causality to determine the causal effect of RST on AMD [8,9]. The hypothesis was that a longer RST contributes to the development of AMD and decreases retinal thickness. As such, limiting RST is expected to be a potential method for preventing AMD.

## METHODS

**Figure 1:**
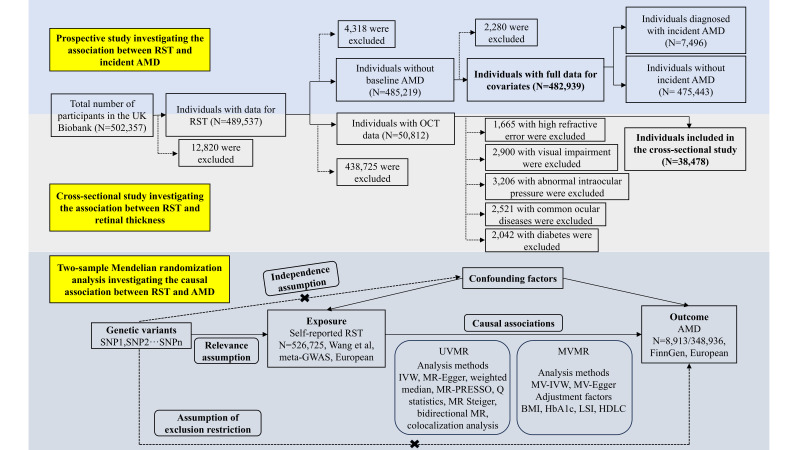
Flowchart of the study design. AMD – age-related macular degeneration, BMI – body mass index, GWAS – genome-wide association study, HbA1c – glycated haemoglobin, HDLC – high-density lipoprotein cholesterol, IVW – inverse variance weighted, LSI – lifetime smoking index, MR – Mendelian randomisation, MV – multivariable, MVMR – multivariable Mendelian randomisation, OCT – optical coherence tomography, PRESSO – pleiotropy residual sum and outlier, RST – recreational screen time, SNP – single nucleotide polymorphisms, UVMR – univariable Mendelian randomisation.

We divided the study into three parts – a prospective cohort study, a cross-sectional study, and a two-sample MR analysis (**Figure 1**; Methods S1 in the **Online Supplementary Document**). Using UK Biobank follow-up data, we first prospectively investigated the association of different durations of RST and incident AMD. We then performed two-sample univariable MR, multivariable MR, and corresponding sensitivity analyses using summary-level genome-wide association study (GWAS) data to estimate the potential causal relationship between RST and AMD. AMD is found to be associated with the changes of total retinal thickness and each layer thickness [10–16], suggesting that RST may play a role in the development of AMD by affecting retinal thickness. Therefore, we explored the association between RST and retinal layer thickness in a cross-sectional way, using OCT data from UK Biobank. We reported the article in accordance with the recommendations of the Strengthening the Reporting of Observational Studies in Epidemiology (STROBE) and STROBE-MR guidelines.

## RESULTS

### Prospective estimates of the association between RST and incident AMD

Our prospective cohort included 482 939 UK Biobank participants with a mean age of 56.47 years (standard deviation (SD) = 8.09 years). 220 856 were male (45.7%). The median follow-up period was 12.8 years, during which 7496 individuals developed AMD. Participants with longer daily RST were more likely to be older, male, non-white, less educated, had a greater Townsend deprivation index, had a history of smoking, and a history of diabetes, cardiovascular disease, hypertension, and hyperlipidaemia (Table S2 in the **Online Supplementary Document**). We also compared the baseline characteristics between participants who did or did not develop AMD during the follow-up period (Table S3 in the **Online Supplementary Document**).

**Figure 2:**
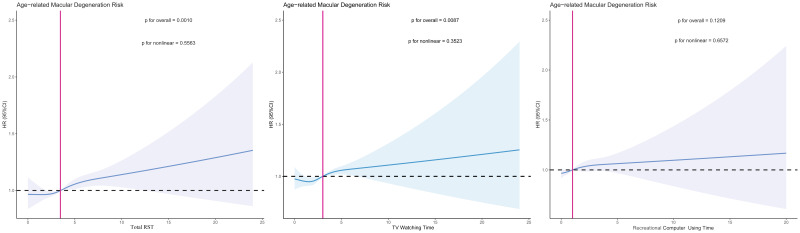
Association between recreational screen time and age-related macular degeneration evaluated by restricted cubic spline curves. The HR (solid blue line) and 95% CI (light blue area) are from a multivariable-adjusted Cox regression model using restricted cubic spline curves with four knots (5th, 35th, 65th, and 95th percentiles). Adjustment includes age, sex, race, education, Townsend deprivation index, smoking status, history of hypertension, hyperlipidaemia, diabetes, and cardiovascular diseases. The red line indicates the point where HR = 1.0. CI – confidence interval, HR – hazard ratio, RST – recreational screen time.

After multivariable adjustment, increases in total daily RST (hazard ratio (HR) per hour = 1.02, 95% confidence interval (CI) = 1.01, 1.03; HR per SD = 1.05, 95% CI = 1.03, 1.07, *P* < 0.001), recreational computer use time (HR per hour = 1.02, 95% CI = 1.00, 1.04; HR per SD = 1.03, 95% CI = 1.00, 1.06, *P* = 0.026), and television watching time (HR per hour = 1.02, 95% CI = 1.01, 1.04; HR per SD = 1.04, 95% CI = 1.02, 1.06, *P* = 0.001) were linked to a greater risk of developing AMD. After transforming the exposure variable into tertiles, high RST (>4 h/d) still increased the risk of AMD compared to low RST (HR = 1.09; 95% CI = 1.04, 1.15, *P* = 0.001), but moderate RST (3.5–4 h/d) did not have a significant impact on AMD onset (**Table 1**). Similar results were obtained when RST was converted to its quintile form (Table S4 in the **Online Supplementary Document**). According to the sensitivity analyses, the association between RST and AMD remained consistent even when we excluded participants diagnosed with AMD within two years of the baseline assessment, when we excluded participants with self-reported AMD diagnoses, or when we included additional covariates in the model (Table S5–7 in the **Online Supplementary Document**). The results of the subgroup analyses remained generally consistent, with no interactions detected (Table S8 in the **Online Supplementary Document**). The association between RST and AMD was found to be linear according to the restricted cubic spline plots; that is, the longer the daily RST was, the greater the risk of AMD (**Figure 2**).

**Table 1:** Prospective estimates of the association between recreational screen time and age-related macular degeneration

	Unadjusted model		Partially-adjusted model*		Fully-adjusted model†	
**Exposure**	**HR (95% CI)**	***P*-value**	**HR (95% CI)**	***P*-value**	**HR (95% CI)**	***P*-value**
**Total daily RST**						
Per hour increase	1.06 (1.05, 1.07)	<0.001	1.04 (1.03, 1.05)	<0.001	1.02 (1.01, 1.03)	<0.001
Per SD increase	1.14 (1.11, 1.16)		1.08 (1.06, 1.11)		1.05 (1.03, 1.07)	
Tertiles						
*Low (0–3 h/d)*	ref		ref		ref	
*Moderate (3.5 4 h/d)*	1.26 (1.19, 1.34)	<0.001	1.06 (1.00, 1.13)	0.042	1.05 (0.98, 1.11)	0.150
*High (>4 h/d)*	1.44 (1.37, 1.52)	<0.001	1.16 (1.10, 1.22)	<0.001	1.09 (1.04, 1.15)	0.001
**Daily recreational computer use time**						
Per hour increase	0.94 (0.93, 0.96)	<0.001	1.02 (1.00, 1.04)	0.035	1.02 (1.00, 1.04)	0.026
Per SD increase	0.92 (0.90, 0.95)		1.03 (1.00, 1.05)		1.03 (1.00, 1.06)	
Tertiles						
*Low (0–3 h/d)*	ref		ref		ref	
*Moderate (3.5–4 h/d)*	0.87 (0.82, 0.92)	<0.001	1.01 (0.95, 1.06)	0.770	1.04 (0.99, 1.10)	0.118
*High (>4 h/d)*	0.92 (0.86, 0.97)	0.003	1.07 (1.01, 1.13)	0.032	1.07 (1.01, 1.14)	0.023
**Daily television-watching time**						
Per hour increase	1.13 (1.12, 1.14)	<0.001	1.04 (1.03, 1.06)	<0.001	1.02 (1.01, 1.04)	0.001
Per SD increase	1.23 (1.21, 1.25)		1.08 (1.05, 1.10)		1.04 (1.02, 1.06)	
Tertiles						
*Low (0–3 h/d)*	ref		ref		ref	
*Moderate (3.5–4 h/d)*	1.33 (1.25, 1.41)	<0.001	1.07 (1.01, 1.13)	0.028	1.04 (0.98, 1.11)	0.155
*High (>4 h/d)*	1.75 (1.66, 1.85)	<0.001	1.18 (1.12, 1.24)	<0.001	1.10 (1.04, 1.16)	0.001

CI – confidence interval, HR – hazard ratio, ref – reference, RST – recreational screen time, SD – standard deviation

*Adjusted for age and sex.

†Adjusted for age, sex, race, education, Townsend deprivation index, smoking status, history of hypertension, hyperlipidaemia, diabetes, and cardiovascular diseases.

### MR estimates of the causal associations between RST and AMD

We removed possible pleiotropic single nucleotide polymorphisms (SNPs) through PhenoScanner (University of Cambridge, Cambridge, UK) and identified 49 independent SNPs as instrumental variables for the univariable MR analysis. These genetic variants explained 0.34% of the total variance in the RST phenotype. All SNPs were strong instruments, as their F-statistics ranged from 29.9–55.1, which is much greater than 10 (Table S11 in the **Online Supplementary Document**).

**Figure 3:**
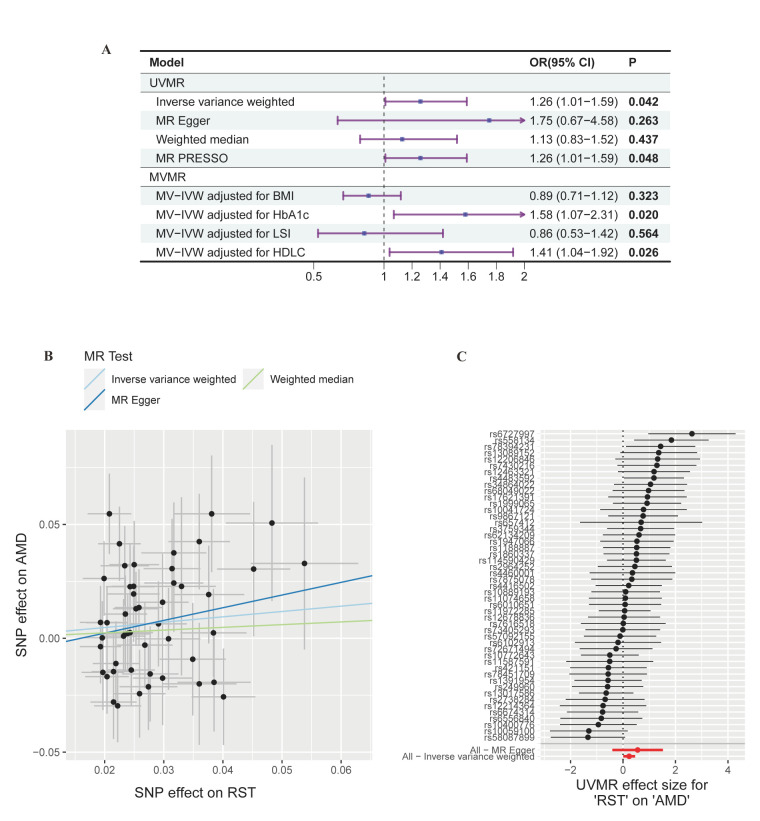
MR estimates of the causal associations between recreational screen time and age-related macular degeneration. **Panel A.** The main results of UVMR and MVMR estimation. **Panel B.** Scatter plot of the UVMR analysis. **Panel C.** Forest plot of the UVMR analysis. AMD – age-related macular degeneration, BMI – body mass index, CI – confidence interval, HbA1c – glycated haemoglobin, HDLC – high-density lipoprotein cholesterol, IVW – inverse variance weighted, LSI – lifetime smoking index, MR – Mendelian randomisation, MVMR – multivariable Mendelian randomisation, OR – odds ratio, PRESSO – pleiotropy residual sum and outlier, RST – recreational screen time, SNP – single nucleotide polymorphisms, UVMR – univariable Mendelian randomisation.

According to the univariable MR results based on the inverse variance weighting (IVW) model (**Figure 3**, Panel A–C; Table S10 in the **Online Supplementary Document**), each increase of one SD in genetically predicted daily RST duration was linked to a 26% increase in the risk of AMD (odds ratio (OR) = 1.26; 95% CI = 1.01, 1.59, *P* = 0.042). The MR-Egger, weighted median and MR Pleiotropy RESidual Sum and Outlier estimates were in the same direction as the IVW estimates, confirming the robustness of the IVW estimates (Table S10 in the **Online Supplementary Document**). We did not observe significant horizontal pleiotropy or heterogeneity according to the Egger intercept (*P* = 0.503) or the Q statistic (*P* = 0.065 for the IVW model and *P* = 0.059 for the MR Egger model). Moreover, reverse MR analysis suggested that AMD has no causal effect on RST, which is consistent with the findings of the MR Steiger directionality test (Table S10 and Table S12 in the **Online Supplementary Document**). Both tests indicated that the causal effect of RST on AMD was in the right direction.

We conducted two sensitivity analyses using different instrument selection criteria, which confirmed our primary findings. First, we adjusted the linkage disequilibrium threshold (r^2^<0.01 within a 5000 kb window), which increased the number of valid SNPs in the analysis set to 54, and subsequently obtained consistent results (OR = 1.30; 95% CI = 1.07, 1.58, *P* = 0.010) (Table S13 in the **Online Supplementary Document**). Second, in the single-gene MR using instrumental variables from the Kinesin Light Chain 2 gene region, the risk effect of RST on AMD was strengthened (OR = 3.70; 95% CI = 1.60, 8.57, *P* = 0.002) (Table S14 in the **Online Supplementary Document**). Additionally, there is no evidence to suggest that the Kinesin Light Chain 2 gene region contains common causal variants for RST and AMD (posterior probability of hypothesis one = 0.862, posterior probability of hypothesis four = 0.067) (Figure S1 and Table S15 in the **Online Supplementary Document**), indicating that the variants in this region are significantly associated with RST and are unlikely to affect the outcome. This finding is consistent with the main assumptions of MR and further supports our MR estimates.

After adjusting for glycated haemoglobin (IVW OR = 1.58; 95% CI = 1.07, 2.31, *P* = 0.020) and high-density lipoprotein cholesterol (IVW OR = 1.41; 95% CI = 1.04, 1.92, *P* = 0.026) separately, the causal relationship between RST and AMD remained (**Figure 3**, Panel A; Table S16 in the **Online Supplementary Document**). However, after adjusting for lifetime smoking index (IVW OR = 0.86; 95% CI = 0.53, 1.42, *P* = 0.564) and body mass index (BMI) (IVW OR = 0.89; 95% CI = 0.71, 1.12, *P* = 0.323), the association between RST and AMD was no longer statistically significant. This suggests that the relationship between RST and AMD could be influenced by factors such as obesity and smoking status. However, this result should be interpreted with caution given that the instrumental strength of RST was reduced in multivariable MR models incorporating BMI or lifetime smoking index (conditional F-value for RST < 10), thus reducing the ability of multivariate MR to estimate causal effects. For this reason, we further conducted subgroup and interaction analyses using the UK Biobank cohort. We found that the effects of RST on AMD were largely consistent across subgroups with different BMI or subgroups with different smoking status. No significant interactions were detected, which supports the idea that the effect of RST on AMD is independent of obesity or smoking status (Table S8 in the **Online Supplementary Document**). The multivariable MR IVW and multivariable MR Egger results were consistent, and the *P*-values of the multivariable MR Egger intercepts were >0.05, indicating that the risk of bias due to horizontal pleiotropy was low (Table S16 in the **Online Supplementary Document**).

### The association between RST and retinal thickness

We included 38 478 participants with valid OCT data in the cross-sectional study. The participants’ mean age was 56.47 years (SD = 8.09), and 220 856 of them were men (45.7%). The general characteristics of the cross-sectional population stratified according to RST were similar to those of the prospective cohort (Table S17 in the **Online Supplementary Document**).

An increase in the total daily RST was associated with thinning of various retinal layers, including the outer retina (retinal pigment epithelium (RPE) and photoreceptor cell layer), inner retina (ganglion cell-inner plexiform layer (GC-IPL) and inner nuclear layer (INL)), and total macular thickness (**Table 2**). Interestingly, the study revealed that daily television watching had a more significant impact on retinal thinning than daily recreational computer use (Table S18–19 in the **Online Supplementary Document**). We also used restricted cubic spline plots to determine the shape of the association between total daily RST and retinal thickness (Figure S2 in the **Online Supplementary Document**).

**Table 2:** Cross-sectional estimates of the association between total daily recreational screen time and retinal thickness

	Unadjusted model		Partially-adjusted model†		Fully-adjusted model‡	
Outcome*	***β* (95% CI)**	***P*-value**	***β* (95% CI)**	***P*-value**	***β* (95% CI)**	***P*-value**
RNFL	–0.17 (–0.21, –0.13)	<0.001	–0.11 (–0.15, –0.07)	<0.001	–0.04 (–0.08, 0.00)	0.071
GC-IPL	–0.19 (–0.24, –0.13)	<0.001	–0.12 (–0.17, –0.06)	<0.001	–0.14 (–0.20, –0.09)	<0.001
INL	0.01 (–0.01, 0.03)	0.519	0.00 (–0.02, 0.02)	0.970	–0.03 (–0.05, –0.01)	0.014
INL-ELM	–0.10 (–0.16, –0.04)	0.002	–0.17 (–0.23, –0.11)	<0.001	–0.16 (–0.22, –0.09)	<0.001
ELM-ISOS	–0.09 (–0.10, –0.07)	<0.001	–0.07 (–0.09, –0.06)	<0.001	–0.05 (–0.06, –0.03)	<0.001
ISOS-RPE	–0.11 (–0.15, –0.07)	<0.001	–0.13 (–0.17, –0.09)	<0.001	–0.07 (–0.11, –0.03)	<0.001
INL-RPE	–0.29 (–0.37, –0.22)	<0.001	–0.37 (–0.45, –0.30)	<0.001	–0.28 (–0.35, –0.20)	<0.001
RPE	–0.01 (–0.04, 0.01)	0.284	–0.01 (–0.04, 0.02)	0.516	–0.05 (–0.08, –0.02)	0.001
Macula	–0.64 (–0.77, –0.52)	<0.001	–0.60 (–0.73, –0.47)	<0.001	–0.49 (–0.61, –0.36)	<0.001

CI – confidence interval, ELM-ISOS – external limiting membrane-inner and outer segments of photoreceptor, GC-IPL – ganglion cell-inner plexiform layer, INL – inner nuclear layer, INL-ELM – inner nuclear layer-external limiting membrane, INL-RPE – inner nuclear layer-retinal pigment epithelium, ISOS-RPE – inner and outer segments of photoreceptor-retinal pigment epithelium, RNFL – retinal nerve fibre layer, RPE – retinal pigment epithelium

*Effect of per standard deviation increase of recreational screen time on the outcomes.

†Adjusted for age and sex.

‡Adjusted for age, sex, race, education, Townsend deprivation index, smoking status, history of hypertension, hyperlipidaemia and cardiovascular diseases, visual acuity of the better eye, mean intraocular pressure and mean spherical equivalent of both eyes.

Given that the cross-sectional design made the results susceptible to reverse causation and residual confounding, we conducted a two-sample MR analysis using available GWAS data to validate the initial observational findings. The results indicated that longer RST was associated with an increased risk of inner retinal thinning (retinal nerve fibre layer (RNFL) and GC-IPL) (Table S20 in the **Online Supplementary Document**), while no association was found with outer retinal thickness (photoreceptor cell layer). Moreover, the reverse MR analysis, in conjunction with the MR Steiger directionality test, suggested that the effect of RST on retinal thickness was more significant than the reverse effect of retinal thickness on RST, supporting the proposed direction of causality (Table S20–21 in the **Online Supplementary Document**).

## DISCUSSION

The impact of RST, an important risk factor in modern society, on health cannot be ignored. Previous studies have mainly explored the associations of RST with ageing, cognition, and neurodegeneration, although the evidence is still insufficient. AMD, a representative ocular neurodegenerative disease, is closely related to cognitive impairment and degeneration of the central nervous system [17,18]. However, the relationship between RST and AMD has not been elucidated. A recent cross-sectional study revealed that a considerable number of elderly AMD patients had a habit of using computers, suggesting an increase in screen use among the elderly population, but did not evaluate the association between screen time and AMD [19]. In this comprehensive study, which combined findings from observational studies and MR analyses, we found that a longer RST may be a potential causal risk factor for AMD. Combining results from these two methods with different sources of bias provides more convincing evidence.

One reason why longer RST increases the risk of AMD and other neurodegenerative diseases may be its adverse effects as a kind of sedentary behaviour. Excessive RST is traditionally considered a form of sedentary behaviour because people hardly fixate on the screen during exercise of greater intensity. Sedentary behaviour, including prolonged television watching and computer use, can lead to neurodegeneration by changing hemodynamics, promoting systemic metabolic disorders, and aggravating neuroinflammatory responses [3,20].

However, evidence supports the role of RST as a risk factor independent of sedentary behaviour, especially for ocular diseases. For example, RST may contribute to developing AMD lesions through retinal light damage. Biological studies have investigated the effects of screen time-related exposures, such as blue light and light-emitting diodes (LEDs), on retinal health. White LEDs, commonly used in electronic screens, produce white light by coupling blue light from blue LEDs with yellow phosphors [21]. Blue light is known to cause toxicity and damage to retinal cells, including photoreceptors, Müller cells, and especially to RPE cells, which play a crucial role in AMD progression [22–25]. For instance, Alaimo et al. found that blue light increases oxidative stress and leads to RPE cell apoptosis [26]. Nakamura et al. reported that blue LEDs damage RPE and photoreceptors in mice, causing drusen-like substance accumulation [27]. Blue light not only damages the outer retina but also contributes to lesions in the inner retina. Long-term exposure to blue light has been shown to significantly thin various layers of the retina in mice, particularly affecting the RPE, outer nuclear layer, and inner retinal layers, such as the inner plexiform layer and INL [28].

Our cross-sectional study also provides some support for the hypothesis that the risk associated with RST on AMD may be mediated through retinal photodamage. We found that RST was significantly linked to the thinning of the outer retina (RPE and photoreceptor cell layer), the inner retina (GC-IPL and INL), as well as overall macular thickness, consistent with findings from several earlier OCT-based AMD studies [10,12,14,16]. However, MR analysis did not fully confirm these observational results. It suggested that RST was only associated with thinning of the inner retina, specifically the retinal nerve fibre layer and GC-IPL, but not the outer retina. This discrepancy might indicate that residual confounders could bias the results of observational studies. However, since the GWAS on retinal thickness was based on a small sample size, weak associations may have been overlooked [29]. Regardless, the MR analysis addressed the cross-sectional study’s limitation of being unable to determine causal direction, suggesting that RST is more likely to affect retinal thickness rather than early AMD changes influencing patients’ screen-use habits. Unfortunately, we could not fully validate the cross-sectional study results due to a lack of available GWAS data related to RPE, INL, and overall macular thickness. Therefore, large prospective cohort studies and large-scale MR studies are warranted to further validate the effect of RST on retinal thickness.

We found that watching television may be more harmful to the retina than using a computer, even though both activities increase the risk of AMD. On the one hand, using a computer provides more information and engages more cognitive processes than watching television. It has been reported that computer use provides more cognitive stimulation, which may reduce the risk of cognitive decline [6,30]. Some studies have even shown that watching television and using computers have completely different effects on neurodegenerative diseases such as dementia and Parkinson disease, and the differences may be caused by different effects on brain structures measured by total brain volume and hippocampal volume [3]. On the other hand, a growing number of elderly individuals are using computers for social engagement, and there is evidence that computer use enhances their sense of social connectedness, reduces feelings of loneliness, and alleviates depression in ways that television watching does not [31]. Although the role of depression in AMD is not clear, studies have shown that depression and other mental health conditions have adverse effects on visual function in AMD patients, independent of ocular lesions [32,33]. Therefore, the risk of AMD and retinopathy from television watching may be more significant from a mental health perspective. Additionally, differences in display characteristics between television and computer screens may contribute to the variability in the risk of AMD. Traditional television screens typically have lower refresh rates and resolutions than computer monitors [34,35]. Higher refresh rates allow smoother image transitions, while higher resolutions enhance image clarity [36]. Although no direct correlation is established between screen display characteristics and AMD, lower refresh rates and resolutions may increase eye fatigue, potentially leading to retinal damage. For instance, Bali et al. noted that low refresh rates can cause images to flicker, making it harder for the eyes to adapt [37]. Meanwhile, Ziefle found that low-resolution screens increase stress on the eyes’ accommodation, both of which can exacerbate eye fatigue [38]. Research suggests that eyestrain may lead to changes in retinal thickness and neuronal degeneration [39]. Therefore, the lower resolution and refresh rate of conventional television screens might indirectly promote AMD by causing fatigue. However, further studies are needed to confirm this relationship and to explore the connection between screen display characteristics and retinal health.

Our study is important because no previous study has explored the association between RST and AMD. We confirmed the risk effect of RST on AMD and provided an explanation for the association between RST and retinal thickness. Furthermore, we revealed that daily RST > 4 h might increase the risk of AMD. These findings further demonstrate the impact of RST on neurodegenerative diseases and are expected to provide new ideas for the prevention and treatment of AMD. However, this study also has limitations. First, the RST in this study was self-reported, which may be subject to recall bias. While experienced nurses validated the self-reported data gathered at the UK Biobank to minimise bias, it is important to acknowledge that self-reported information has inherent limitations compared to objectively measured data. Therefore, future investigations into the daily screen usage habits of study participants should utilise technologies such as electronic monitoring to further validate their impact on AMD, conditions permitting. Second, the cohort study found relatively low exposure effect values, even with a large sample size. This may be attributed to limitations in the UK Biobank data, which hinder the accurate assessment of participants’ long-term RST profiles, especially as their habits may have changed over time. Nevertheless, our MR analyses used genetic variants linked to lifetime screen-use habits, revealing significantly larger effect sizes and highlighting the importance of managing RST to prevent AMD. However, the relationship between genetic variation and exposure can vary throughout a person’s life, so the estimated “lifetime effect” of exposure from MR should be interpreted cautiously [40]. Further research is necessary to explore how changes in screen use habits might influence AMD risk in the future. Third, the study population in this study was predominantly European, so our results may not be generalisable to other populations. Fourth, due to limitations in the data available from the UK Biobank, we were unable to obtain information about participants' screen use at work. Consequently, this study primarily focuses on RST, which may limit our understanding of the overall impact of screen time on AMD. Since screen use habits may differ between working and retired populations, future research should further explore the relationship between work-related screen use and AMD, as well as compare it to recreational screen use. Fifth, the close association of RST with sedentary behaviour has limited our ability to explore its independent effects on AMD. With the advancement of technology, it may be possible to study the effects of RST and other sedentary behaviours separately in the future, such as by using virtual reality technology to explore the relationship between RST and AMD during exercise. Sixth, the overall genetic interpretability of the IVs was relatively low, which may have restricted our ability to make causal inferences using MR. This is partly due to our stringent selection criteria for instrumental variables, which resulted in a smaller number of instruments. Furthermore, the complexity of the RST phenotype means that current GWAS provide only limited insights into its variations. Although we have conducted several sensitivity analyses to verify the robustness of our findings, there is still a need for larger, cross-ethnic GWASs in the future to better uncover the genetic basis of RST.

## CONCLUSIONS

In conclusion, in this study, we demonstrated through a prospective cohort and MR evidence that longer RST may be a potential causal risk factor for AMD. Reducing RST could be beneficial for preventing AMD, and future research should identify the most effective time points for intervening on RST.

## Additional material


Online Supplementary Document
