# Supplementary material for: Longer recreational screen time contributes to the risk of age-related macular degeneration: a UK Biobank cohort study and two-sample Mendelian randomisation
Source: J Glob Health. 2025 Jan 31;15:04029. doi: 10.7189/jogh.15.04029 (PMC11781809; doi:10.7189/jogh.15.04029)
Supplement: Online Supplementary Document [file jogh-15-04029-s001.pdf]

Supplement to: Huang Y, Zhang X, Liang Li, Jiang Y, Li B, Zhu X, Li C, Gu C, Zou W, Zheng Z, Zhao S. Longer recreational screen time contributes to the risk of age-related macular degeneration: a UK Biobank cohort study and two-sample Mendelian randomisation. J Glob Health. 2025;15:04029.

Online Supplementary Document

Longer recreational screen time contributes to the risk of age-related macular degeneration: A UK Biobank cohort study and two-sample Mendelian randomization

Authors: Yikeng Huang, MD[1*], Xinyu Zhang, MD[1*], Li Liang[1*], Yujin Jiang, MS[1], Bo Li, MD[1,5], Xinyu Zhu, PhD[1], Chenxin Li, PhD[1], Chufeng Gu, PhD[1], Wenjun Zou, PhD[4†], Zhi Zheng, MD, PhD[1,2,3†], Shuzhi Zhao, MD, PhD [1†]

Institutional affiliation:

1. Department of Ophthalmology, Shanghai General Hospital, Shanghai Jiao Tong University School of Medicine; National Clinical Research Center for Eye Diseases; Shanghai Clinical Research Center for Eye Diseases; Shanghai Key Clinical Specialty; Shanghai Key Laboratory of Ocular Fundus Diseases; Shanghai Engineering Center for Visual Science and Photomedicine; Shanghai engineering center for precise diagnosis and treatment of eye diseases; 100 Haining Road, Hongkou District, Shanghai 200080, China.
2. Ningde Municipal Hospital of Ningde Normal University, Fujian Medical University; 13 Mindong East Road, Dongqiao Economic and Technological Development Zone, Ningde City, Fujian Province 352100, China.
3. Fujian Medical University, 1 Xuefu North Road, University New District, Fuzhou City, Fujian Province 350122, China.
4. Wuxi No.2 People's Hospital, Jiangnan University Medical Center; 68 Zhongshan Road, Liangxi District, Wuxi City, Jiangsu Province 214002, China.
5. Department of Ophthalmology, the Fourth Affiliated Hospital of Soochow University, 9 Chongwen Road, Suzhou Industrial Park, Suzhou city 215123, Jiangsu Province, China.

* Yikeng Huang, Xinyu Zhang and Li Liang contributed equally to this work.

† **Corresponding author:** Wenjun Zou, Zhi Zheng and Shuzhi Zhao.

Wenjun Zou **(Co-corresponding author)**:

Address: No. 68 Zhongshan Road, Liangxi District, Wuxi City, Jiangsu Province, China.

Telephone number: 86-0510-68562222; Email address: wenjunzou2022@163.com.

Zhi Zheng **(Co-corresponding author)**:

Address: No. 100 Haining Road, Hongkou District, Shanghai, China.

Telephone number: 86-21-63240090; Email address: zzheng88@sjtu.edu.cn.

Shuzhi Zhao **(Primary corresponding author)**:

Address: No. 100 Haining Road, Hongkou District, Shanghai, China.

Telephone number: 86-21-63240090; Email address: wellsnow2008@163.com.

Table of Contents

**Supplementary Methods.**	……..…....5
**1.** UK Biobank cohort and study population	……..…....5
**2.** Retinal OCT and retinal layer segmentation	……..…....5
**3.** Ascertainment of RST, AMD and covariates	……..…....7
**4.** Statistical analysis	……..…....8
**5.** Summary-level GWAS data sources	……..…....9
**6.** Genetic IV selection	………....11
**7.** MR analysis	………....12
**Table S1** Details of covariates in the UK Biobank study	………....17
**Table S2** Baseline characteristics of participants in the prospective cohort analysis stratified by tertiles of RST	………....18
**Table S3** Baseline characteristics of participants in the prospective cohort analysis stratified according to the presence or absence of incident AMD	………....19
**Table S4** Prospective estimates of the association between quintile form of total daily RST and age-related macular degeneration	………....20
**Table S5** Sensitivity analysis excluding participants with an AMD diagnosis record within 2 years of the baseline assessment	………....21
**Table S6** Sensitivity analysis excluding participants with self-reported diagnoses of AMD	………....22
**Table S7** Sensitivity analysis with additional adjustment	………....23
**Table S8** Subgroup and interaction analysis for prospective estimates of the association between RST and AMD	………....24
**Table S9** Information on GWAS datasets in the MR study	………....25
**Table S10** Forward UVMR estimating the association of RST and AMD	………....26
**Table S11** List of 49 conditionally independent genetic variants used as instruments of RST in the UVMR analysis	………....27

**Table S12** Reverse UVMR estimating the association of RST and AMD	…………....28
**Table S13** UVMR estimating the association of RST and AMD using instrumental variables with relaxing linkage disequilibrium threshold	…………....29
**Table S14** UVMR estimating the association of RST and AMD using instrumental variables located in the KLC2 gene region	…………....30
**Table S15** Colocalization analysis of recreational screen time and AMD in the KLC2 gene region	…………....31
**Table S16** MVMR estimating the association of RST and AMD with adjustment for potential confounders	…………....32
**Table S17** General characteristics of participants in the cross-sectional analysis stratified by tertiles of RST	…………....33
**Table S18** Cross-sectional estimates of the association between daily recreational computer using time and retinal thickness	…………....34
**Table S19** Cross-sectional estimates of the association between daily TV watching time and retinal thickness	…………....35
**Table S20** Forward UVMR estimating the association of recreational screen time and retinal thickness	…………....36
**Table S21** Reverse UVMR estimating the association of recreational screen time and retinal thicknesss	…………....37
**Figure S1** SNP regional association plot of RST and AMD in the KLC2 gene region	…………....38
**Figure S2** Association between total daily RST and retinal thickness evaluated by restricted cubic spline curves	…………....39

Supplementary Methods

1. UK Biobank cohort and study population

UK Biobank is a population-based cohort study that recruited approximately 500,000 individuals aged 40 to 69 years from 2006 to 2010 at 22 assessment centers in England, Scotland and Wales[1]. During their visit to the assessment center, participants were asked to complete a questionnaire, undergo physical measurements, and provide a biosample for baseline data. The study also links the participants' health-related records to enable long-term follow-up of their illness experiences.

In the prospective study, only participants with recreational screen time (RST) information and without age-related macular degeneration (AMD) at baseline were included, and those with incomplete covariate data were excluded (**Figure 1**).

2. Retinal OCT and retinal layer segmentation

Optical coherence tomography (OCT) image processing and retinal layer segmentation were carried out using a previously validated protocol[2,3]. The UK Biobank performed spectral-domain OCT imaging (Data-Category 100079) using Topcon 3D OCT 1000 Mk2 (Topcon Inc, Oakland, New Jersey, USA). Images were acquired in a dark room without mydriasis. The macular area was scanned using a three-dimensional volume (512 horizontal A-scan/B-scan; 128 B-scan in a 6 x 6 mm raster pattern). We then used the Topcon Advanced Boundary Segmentation (TABS) algorithm (version 1.6.1.1) to automatically detect the inner and outer boundaries of the retina and each layer[4]. These include the inner limiting membrane (ILM), retinal nerve fiber layer (RNFL), ganglion cell layer (GCL), inner plexiform layer (IPL), inner

nuclear layer (INL), outer limiting membrane (OLM), inner and outer photoreceptor segments (ISOS), retinal pigment epithelium (RPE), and Bruch’s membrane (BM)[2,3]. The algorithm's accuracy and repeatability have been validated by several large studies[4-6]. After segmentation processing, we performed quality control according to the image quality score and three segmentation indicators, including the ILM indicator, validity count indicator, and motion indicators[2,3]. Individuals with an image quality score lower than 45 or the poorest 20% quality of any segmentation indicator were excluded from the analysis set. The image quality score reflects the strength of the scanning signal. The ILM index shows the minimum localized edge strength around the ILM boundary during scanning and can help identify blinks, severe signal fading, and segmentation errors. The validity count indicator is used to identify scans with significant clipping in the z-axis dimension of OCT scans. The motion indicators use both the RNFL and the full retinal thicknesses to calculate Pearson correlations and absolute differences between the thickness data from each set of consecutive B-scans. The resulting indicator scores are determined by the lowest correlation and the highest absolute difference, which help to identify blinks, eye motion artifacts, and segmentation failures.

For our cross-sectional study that used OCT imaging, we first selected participants who had ST information and OCT data. If data were available for both eyes, the mean value for both eyes was calculated. If data were available for only one eye, the data from that eye were used. Next, we excluded participants who had (1) a history of high refractive error greater than 6D or less than -6D, (2) visual impairment with visual

acuity less than 20/30 (Snellen equivalent), (3) abnormal intraocular pressure with intraocular pressure (IOP) ≥22mmHg or ≤5mmHg, (4) common ocular diseases including diabetic retinopathy, cataract, AMD and glaucoma, or (5) diabetes (**Figure 1**).

3. Ascertainment of RST, AMD and covariates

We used self-reported data on RST as exposure, which included the daily time spent watching TV and using the computer for recreational purposes, and their combined duration[7,8]. The UK Biobank provided the data on time spent watching TV (Data-Field 1070) and using the computer for recreational purposes (Data-Field 1080) through related questions in the touchscreen questionnaire. To assess TV watching time, the following question was asked: " In a typical DAY, how many hours do you spend watching TV?" To determine daily recreational computer using time, the following question was asked: " In a typical DAY, how many hours do you spend using the computer? (Do not include using a computer at work)" Participants entered their responses directly on the screen. If the answer was less than 0 hours or more than 24 hours, it was rejected. Those who answered "Do not know" or "Prefer not to answer" were marked as missing data. Responses of "Less than an hour a day" were recoded to 0.5 hours per day. We calculated the total daily RST by adding the time spent watching TV and using the computer for recreational purposes.

Our identification of AMD as an outcome event was based on the following sources[6]: (1) hospital inpatient records of diagnosis (Data-Fields 41270 and 41271) where cases of AMD were identified by the International Classification of Diseases edition 10 (ICD-10) code H353 and ICD-9 code 3625; (2) self-reported history of AMD

collected through the touch-screen questionnaire (Data-Field 6148); (3) medical history obtained through an oral interview with a trained nurse (Field ID 20002), with code 1528 for AMD. Incident AMD cases were defined as individuals who were initially diagnosed with AMD after enrollment.

Covariates in the UK Biobank were gathered through standardized questionnaires, physical measurements, and blood analysis for biomarkers. In the fully-adjusted cox model, we included the following covariates according to the accepted clinical guidelines and published studies[9,10]: sex, age at recruitment, race, educational attainment (college and above or other), socioeconomic status (Townsend Deprivation index, with larger values showing more material deprivation), smoking status (current, previous, never), presence of hypertension, hyperlipidemia, diabetes, and cardiovascular diseases. We additionally adjusted for visual acuity of the better eye, mean intraocular pressure and mean spherical equivalent of both eyes in the fully-adjusted linear model, whereas the history of diabetes was not adjusted because individuals with diabetes were first excluded. Details regarding covariates are provided in **Table S1**.

4. Statistical analysis

We used Cox proportional-hazards models and linear regression models, respectively, to assess the effect of RST on AMD and retinal layer thickness. We constructed three models for different levels of adjustment in the primary analysis: (1) no adjustment model; (2) partially adjusted model for age and gender; (3) fully adjusted models for all covariates described above. To test the robustness of our findings, we performed several

sensitivity analyses: (1) transformation of the continuous measure of RST into its categorical forms of tertiles (low RST [0-3 hours/day], moderate RST [3.5-4 hours/day], and high RST [>4 hours/day]) and quintiles (0-2, 2.5-3, 3.5-4, 4.5-5, >5 hours/day); (2) exclusion of participants with an AMD diagnosis record of less than 2 years after the baseline assessment; (3) exclusion of participants with self-reported diagnoses of AMD to avoid interference by recall bias; (4) further inclusion of covariates such as BMI, diet, alcohol consumption, physical activity, use of antidiabetic, antihypertensive and antilipemic medications, and a polygenic risk score for AMD. Subgroup analyses were performed based on demographic and lifestyle factors such as age, gender, employment status, smoking status, and obesity status, and likelihood ratio tests were used to assess multiplicative scale interactions. Moreover, we used restricted cubic spline (RCS) plots with four knots at the 5th, 35th, 65th, and 95th percentiles to flexibly model the relationship between RST and AMD. For the Cox model, we tested the proportional hazards assumption using the Schoenfeld residual, and found no violations of this assumption in our analysis. We reported hazard ratios (HRs) and their corresponding 95% confidence intervals (CIs) to reflect effect sizes. For the linear model, we calculated β values and 95% CIs accordingly. All P-values were two-sided with a $P < 0.05$ indicating statistical significance. We used Stata (version 13; StataCorp LLC, College Station, Texas USA) and R software (version 4.2.1; the R Foundation for Statistical Computing, Vienna, Austria) to perform the above statistical analysis.

5. Summary-level GWAS data sources

Data sources were selected from publicly available genome-wide association study

(GWAS) summary data, and details about the various GWAS datasets are shown in **Table S11**.

We extracted the RST-related summary data from the latest and largest meta-GWAS[11]. To avoid bias due to different ethnic groups, only data from European populations were selected for analysis. This meta-GWAS identified 88 loci associated with RST based on 23 cohorts of 526,725 individuals of European ancestry. Details regarding genotyping profiles, phenotypic definitions, and analytical methods for each cohort have been provided in the corresponding study[11].

Summary data on AMD were extracted from the R9 release of the FinnGen consortium, and contained 357,849 individuals of European ancestry with 8,913 cases and 348,936 controls[12]. FinnGen established diagnoses of relevant conditions on the basis of the hospital diagnosis codes and cause of death codes of ICD 8-10 and the surgical and procedural codes of the Nordic Medical Statistics Commission (NOMESCO). The definition of AMD includes both dry and wet AMD. Genetic associations were adjusted for age, sex, 10 principal components, and genotyping batch. More details on FinnGen and the specific diagnostic codes used are available through *https://www.finngen.fi/en/researchers/clinical-endpoints*. The maximum overlap between GWAS of exposure and outcome was not more than 0.5%, so the risk of bias due to sample overlap was considered to be acceptable[13].

Summary-level data on body mass index (BMI) was from the Genetic Investigation of Anthropometric Traits consortium (GIANT) (N = 806,834)[14], data on glycated hemoglobin (HbA1c) (N = 361,194)[15] and lifetime smoking index (LSI) (N =

462,690)[16] was from the UK Biobank, and data on high-density lipoprotein cholesterol (HDLC) was from the Global Lipids Genetics Consortium (GLGC) (N = 1,320,016)[17]. Data on outer (including inner segment layer and outer segment layer of photoreceptor cells, N = 31,135) and inner retinal thickness (including RNFL and ganglion cell-inner plexiform layer, N = 31,434) were obtained from two studies based on UK Biobank by Currant et al[18,19].

6. Genetic IV selection

We selected single nucleotide polymorphisms (SNPs) associated with RST from the exposure GWAS dataset as instrumental variables (IVs), using a genome-wide significance threshold ($P < 5 \times 10^{-8}$). To ensure that these IVs were independent, we removed variants at the same locus based on linkage disequilibrium (LD) ($r^2 < 0.001$ within a 10,000 kb window). We calculated R^2 and F-statistics using established formulas, and empirically excluded SNPs with F-statistics <10 to avoid bias from weak IVs[20]. SNPs with a minimum allele frequency (MAF) < 0.01 were also removed, which left only common variants for analysis. We then harmonized the effects of IVs on exposure and outcome to ensure that the reported effects were for the same allele. This process simultaneously excluded palindromic SNPs and SNPs with incompatible alleles. We also excluded SNPs with P-outcome values lower than the nominal P-value after bonferroni correction (P < 0.05/N.SNPs) as they were considered directly related to the outcome trait[21]. To minimize potential pleiotropic effects from confounders, we excluded SNPs associated with other traits using PhenoScanner V2 (P-confounder $< 5 \times 10^{-8}$, www.phenoscanner.medschl.cam.ac.uk, accessed on 06 December

2023)[22]. We obtained IVs for multivariable Mendelian randomization (MVMR) analysis by combining BMI, HbA1c, HDLC-associated SNPs and RST-associated SNPs separately for mutual adjustment. The details of the SNPs included in the univariable Mendelian randomization (UVMR) analysis set are shown in **Table S13.**

Furthermore, we used two additional IV selection criteria for UVMR as a supplementary analysis: (1) To increase the statistical power of our analysis, we included more SNPs by moderately relaxing the LD threshold (r2 < 0.01 within a 5,000 kb window). (2) Wang and colleagues used seven different methods to identify potential genes in RST-associated loci, and found that the KLC2 gene was prioritized by up to three of these methods simultaneously[11]. As a result, KLC2 is considered to be one of the most likely genes to reflect the genetic mechanism of RST. Therefore, we selected SNPs located in the KLC2 gene region (chr11:66,024,759-66,035,331, GRCh37/hg19 by NCBI) as IVs and excluded SNPs in LD with an $r^2 > 0.80$ for single-gene Mendelian randomization (MR) to minimize pleiotropy[23].

7. MR analysis

In order to perform MR analyses, IVs must satisfy the following three major assumptions[24]:(1) Relevance assumption: genetic variants included in the analysis must be strongly associated with the exposure of interest; (2) Independence assumption: the variants should not be associated with confounders that may influence the exposure-outcome relationship; (3) Assumption of exclusion restriction: the variants included should affect the outcome solely through the exposure. For a better understanding, please refer to **Figure 1**.

We first conducted UVMR to examine the causal relationship between genetically predicted RST and AMD. Our primary method was inverse variance weighting (IVW) with multiplicative random effects, which summarizes the Wald ratio estimates of the causal effects of each SNP, assuming that all IVs are valid, while considering potential heterogeneity among SNPs[25]. To account for horizontal pleiotropy and ensure the robustness of the findings, we also used three additional methods with more relaxed assumptions about IVs, namely MR-Egger[26], weighted median[27], and Mendelian Randomization Pleiotropy RESidual Sum and Outlier (MR-PRESSO)[28] methods. The MR-Egger method, under the Instrument Strength Independent of Direct Effect (InSIDE) assumption, tests for horizontal pleiotropy and provides pleiotropy-corrected causal estimates[26]. Although this method is usually underpowered, the non-zero intercepts can indicate the direction of pleiotropy[29]. The weighted median approach is considered to give accurate causal estimates, even when half of the SNPs are null[27]. MR-PRESSO, on the other hand, identifies outlier SNPs that introduce pleiotropy and generates estimates after excluding them[28]. We also used the Q-statistic to assess heterogeneity among IVs, with Q-P values > 0.05 indicating no significant heterogeneity[25]. Furthermore, we applied the MR Steiger directionality test and bidirectional MR to clarify the causal direction[30,31]. For single-gene MR, we performed a colocalization analysis using a Bayesian model to investigate whether RST and AMD share common causal variants in a given region[32]. In brief, we extracted genetic variants within 200 kb of the corresponding gene and calculated the posterior probability (PP). A PP.H4 of 0.80 or higher was considered as evidence of positive

colocalization[32].

MVMR analysis is an extension of UVMR by incorporating SNPs associated with multiple exposures to simultaneously detect the direct causal effect of each exposure on outcomes[33]. When adjusting for confounders or potentially pleiotropic pathways, MVMR provides an unbiased estimate of the causal effect of exposure on the outcome[34]. Previous studies have shown that there is a significant link between RST and certain health issues, such as high BMI, high blood glucose, and low HDLC[11]. Additionally, smoking and blood lipid levels are also known to be potential risk factors for AMD[10]. Therefore, these factors could affect the relationship between RST and AMD, and we used MVMR to account for them. Similarly, we used IVW as the main MVMR analysis method. We also applied the MVMR Egger method to verify the robustness of the IVW results in the MVMR analysis.

We assessed the strength of causal effects using the odds ratios (ORs) and corresponding 95% CIs. All P-values were two-sided and a conventional significance level (P-value < 0.05) was used. All MR analyses were conducted using R packages “TwoSampleMR” (version 0.5.6), “MRPRESSO” (version 1.0), “Mendelian Randomization” (version 0.6.0) and "MVMR" (version 0.3).

References：

1 Sudlow C, Gallacher J, Allen N, Beral V, Burton P, Danesh J, et al. UK biobank: an open access resource for identifying the causes of a wide range of complex diseases of middle and old age. PLoS Med. 2015;12:e1001779.

2 Ko F, Foster PJ, Strouthidis NG, Shweikh Y, Yang Q, Reisman CA, et al. Associations with Retinal Pigment Epithelium Thickness Measures in a Large Cohort: Results from the UK Biobank. Ophthalmology. 2017;124:105-17.

3 Patel PJ, Foster PJ, Grossi CM, Keane PA, Ko F, Lotery A, et al. Spectral-Domain Optical Coherence Tomography Imaging in 67 321 Adults: Associations with Macular Thickness in the

UK Biobank Study. Ophthalmology. 2016;123:829-40.

4 Yang Q, Reisman CA, Wang Z, Fukuma Y, Hangai M, Yoshimura N, et al. Automated layer segmentation of macular OCT images using dual-scale gradient information. Opt Express. 2010;18:21293-307.

5 Chua SYL, Warwick A, Peto T, Balaskas K, Moore AT, Reisman C, et al. Association of ambient air pollution with age-related macular degeneration and retinal thickness in UK Biobank. Br J Ophthalmol. 2022;106:705-11.

6 Zekavat SM, Sekimitsu S, Ye Y, Raghu V, Zhao H, Elze T, et al. Photoreceptor Layer Thinning Is an Early Biomarker for Age-Related Macular Degeneration: Epidemiologic and Genetic Evidence from UK Biobank OCT Data. Ophthalmology. 2022;129:694-707.

7 Celis-Morales CA, Lyall DM, Steell L, Gray SR, Iliodromiti S, Anderson J, et al. Associations of discretionary screen time with mortality, cardiovascular disease and cancer are attenuated by strength, fitness and physical activity: findings from the UK Biobank study. BMC Med. 2018;16:77.

8 Wu H, Gu Y, Du W, Meng G, Wu H, Zhang S, et al. Different types of screen time, physical activity, and incident dementia, Parkinson's disease, depression and multimorbidity status. Int J Behav Nutr Phys Act. 2023;20:130.

9 Han X, Lee SS, Ingold N, McArdle N, Khawaja AP, MacGregor S, et al. Associations of sleep apnoea with glaucoma and age-related macular degeneration: an analysis in the United Kingdom Biobank and the Canadian Longitudinal Study on Aging. BMC Med. 2021;19:104.

10 Guymer RH, Campbell TG. Age-related macular degeneration. Lancet. 2023;401:1459-72.

11 Wang Z, Emmerich A, Pillon NJ, Moore T, Hemerich D, Cornelis MC, et al. Genome-wide association analyses of physical activity and sedentary behavior provide insights into underlying mechanisms and roles in disease prevention. Nat Genet. 2022;54:1332-44.

12 Kurki MI, Karjalainen J, Palta P, Sipilä TP, Kristiansson K, Donner KM, et al. FinnGen provides genetic insights from a well-phenotyped isolated population. Nature. 2023;613:508-18.

13 Burgess S, Davies NM, Thompson SG. Bias due to participant overlap in two-sample Mendelian randomization. Genet Epidemiol. 2016;40:597-608.

14 Pulit SL, Stoneman C, Morris AP, Wood AR, Glastonbury CA, Tyrrell J, et al. Meta-analysis of genome-wide association studies for body fat distribution in 694 649 individuals of European ancestry. Hum Mol Genet. 2019;28:166-74.

15 Pan-UKB team. 2020. Available: https://pan.ukbb.broadinstitute.org. Accessed.

16 Wootton RE, Richmond RC, Stuijfzand BG, Lawn RB, Sallis HM, Taylor GMJ, et al. Evidence for causal effects of lifetime smoking on risk for depression and schizophrenia: a Mendelian randomisation study. Psychol Med. 2020;50:2435-43.

17 Graham SE, Clarke SL, Wu KH, Kanoni S, Zajac GJM, Ramdas S, et al. The power of genetic diversity in genome-wide association studies of lipids. Nature. 2021;600:675-9.

18 Currant H, Fitzgerald TW, Patel PJ, Khawaja AP, Webster AR, Mahroo OA, et al. Sub-cellular level resolution of common genetic variation in the photoreceptor layer identifies continuum between rare disease and common variation. PLoS Genet. 2023;19:e1010587.

19 Currant H, Hysi P, Fitzgerald TW, Gharahkhani P, Bonnemaijer PWM, Senabouth A, et al. Genetic variation affects morphological retinal phenotypes extracted from UK Biobank optical coherence tomography images. PLoS Genet. 2021;17:e1009497.

20 Papadimitriou N, Dimou N, Tsilidis KK, Banbury B, Martin RM, Lewis SJ, et al. Physical

activity and risks of breast and colorectal cancer: a Mendelian randomisation analysis. Nat Commun. 2020;11:597.

21 Song X, Wang C, Wang T, Zhang S, Qin J. Obesity and risk of gestational diabetes mellitus: A two-sample Mendelian randomization study. Diabetes Res Clin Pract. 2023;197:110561.

22 Kamat MA, Blackshaw JA, Young R, Surendran P, Burgess S, Danesh J, et al. PhenoScanner V2: an expanded tool for searching human genotype-phenotype associations. Bioinformatics. 2019;35:4851-3.

23 Benn M, Nordestgaard BG, Frikke-Schmidt R, Tybjærg-Hansen A. Low LDL cholesterol, PCSK9 and HMGCR genetic variation, and risk of Alzheimer's disease and Parkinson's disease: Mendelian randomisation study. Bmj. 2017;357:j1648.

24 Davies NM, Holmes MV, Davey Smith G. Reading Mendelian randomisation studies: a guide, glossary, and checklist for clinicians. Bmj. 2018;362:k601.

25 Bowden J, Del Greco MF, Minelli C, Davey Smith G, Sheehan N, Thompson J. A framework for the investigation of pleiotropy in two-sample summary data Mendelian randomization. Stat Med. 2017;36:1783-802.

26 Bowden J, Davey Smith G, Burgess S. Mendelian randomization with invalid instruments: effect estimation and bias detection through Egger regression. Int J Epidemiol. 2015;44:512-25.

27 Bowden J, Davey Smith G, Haycock PC, Burgess S. Consistent Estimation in Mendelian Randomization with Some Invalid Instruments Using a Weighted Median Estimator. Genet Epidemiol. 2016;40:304-14.

28 Hartwig FP, Davey Smith G, Bowden J. Robust inference in summary data Mendelian randomization via the zero modal pleiotropy assumption. Int J Epidemiol. 2017;46:1985-98.

29 Burgess S, Bowden J, Fall T, Ingelsson E, Thompson SG. Sensitivity Analyses for Robust Causal Inference from Mendelian Randomization Analyses with Multiple Genetic Variants. Epidemiology. 2017;28:30-42.

30 Hemani G, Tilling K, Davey Smith G. Orienting the causal relationship between imprecisely measured traits using GWAS summary data. PLoS Genet. 2017;13:e1007081.

31 Zheng J, Baird D, Borges MC, Bowden J, Hemani G, Haycock P, et al. Recent Developments in Mendelian Randomization Studies. Curr Epidemiol Rep. 2017;4:330-45.

32 Giambartolomei C, Vukcevic D, Schadt EE, Franke L, Hingorani AD, Wallace C, et al. Bayesian test for colocalisation between pairs of genetic association studies using summary statistics. PLoS Genet. 2014;10:e1004383.

33 Burgess S, Thompson SG. Multivariable Mendelian randomization: the use of pleiotropic genetic variants to estimate causal effects. Am J Epidemiol. 2015;181:251-60.

34 Sanderson E, Davey Smith G, Windmeijer F, Bowden J. An examination of multivariable Mendelian randomization in the single-sample and two-sample summary data settings. Int J Epidemiol. 2019;48:713-27.

Table S1 Details of covariates in the UK Biobank study

Covariates	Type	Data fields or Categories	Description
Age	Integer (years)	Field 21022	A derived variable based on date of birth and date of attending an initial assessment centre and refers to the age of the participant on the day they attended an Initial Assessment Centre, truncated to whole year.
Sex	Categorical (female or male)	Field 31	Sex of participants was aquired from central registry at recruitment, but in some cases updated by the participant.
Ethnicity	Categorical (white or other)	Field 21000	Aquired from the touchscreen questionnaire during the initial Assessment Centre visit.
Educational background	Categorical (college/university or lower)	Field 6138	Aquired from the touchscreen questionnaire during the initial Assessment Centre visit.
Townsend index	Continuous	Field 22189	The Townsend index is an area-based index composite of the percentage of owner-occupied homes, unemployment, car ownership, and crowdedness. A positive value denotes greater material deprivation. Townsend index of participants was calculated immediately prior to participant joining the UK Biobank based on the preceding national census output areas. Each participant is assigned a score corresponding to the output area in which their postcode is located.
Smoking status	Categorical (previous/current or never)	Field 20116	Aquired from the touchscreen questionnaire during the initial Assessment Centre visit.
History of hypertension(1)	Categorical (yes or not)	Fields 93, 4080, 94, 4079, 20003, 6153, 6177, 20002	A history of hypertension was defined as ‘yes’ if one was on antihypertensive medication, reported prior doctor-diagnosed hypertension, or had a blood pressure >= 140/90 mmHg at baseline.
History of hyperlipidemia(2)	Categorical (yes or not)	Fields 30690, 30870, 30760, 30780, 20003, 6153, 6177, 20002	A history of hyperlipidemia was defined as 'yes' if one was on lipid-lowering medication, reported prior doctor-diagnosed hyperlipidemia, had a plasma total triglycerides > 2.30 mmol/L, or had a plasma total cholesterol > 6.21 mmol/L, or had a plasma low-density lipoprotein cholesterol > 4.10 mmol/L, or had a plasma high-density lipoprotein cholesterol < 1.00 mmol/L (male) or < 1.30 mmol/L (female).
History of diabetes(3-4)	Categorical (yes or not)	Fields 41270, 41280, 41271, 41281, 130706, 130708, 130710, 130712, 130714, 6153, 6177, 20002, 20003, 20009, 2443, 30750	A history of diabetes was defined as 'yes' if one was on hypoglycemic medication, with hospital inpatient or self-reported records of diabetes, or had a plasma glycated haemoglobin > 6.5%.
History of cardiovascular diseases(5-7)	Categorical (yes or not)	Fields 42000, 41270, 41280, 41271, 41281, 41272, 41282, 20002, 20004, 20009, 20011, 3894, 3627	A history of cardiovascular diseases was defined as 'yes' if one had any of the disease records including myocardial infarction, angina pectoris, unstable angina, cardiac arrest and chronic ischemic disease, or the treatment records including percutaneous coronary intervention and coronary artery replacement.
Body mass index	Continuous (SD)	Field 21001	Body mass index value is constructed from height and weight measured during the initial Assessment Centre visit, as weight (kg) divided by the square of height (m2).
Diet(10)	Categorical (healthy or unhealthy)	Category 1004	Dietary quality was defined considering adequate consumption of fruit, vegetables, whole grains, fish, shellfish, dairy products, and vegetable oils and reduced consumption of refined grains, processed meats, unprocessed meats, and sugar sweetened beverages. A healthy diet was defined as meeting at least five items of recommendations among these 10 categories.
Alcohol consumption(11)	Continuous (SD)	Category 100051	Alcohol consumption was defined according to self-reported weekly or monthly alcohol consumption in questionnaire. A pint or can of beer/lager/cider: 2 units; a 25ml single shot of spirits: 1unit; a standard glass of wine (175ml): 2 units.
Physical activity	Continuous (SD)	Field 22040	Physical activity was measured in minutes by total metabolic equivalent tasks (METs) performed per week for all activities including walking, moderate intensity, and vigorous activity.
Use of antidiabetic medication	Categorical (yes or not)	Field 20003, 6153, 6177	Self-reported use of insulin or oral diabetic medication, including metformin, glucophage, rosiglitazone, glibenclamide, gliclazide, diamicron, glimepiride, amaryl, glipizide, minodiab, tolbutamide, euglucon, acarbose, glucobay, glucotard, nateglinide, starlix, repaglinide, pioglitazone, actos, troglitazone, rosiglitazone, and avandia.
Use of antihypertensive medication	Categorical (yes or not)	Field 20003, 6153, 6177	Self-reported use of antihypertensive medication, including methyldopa, clonidine, moxonidine, physiotens, prazosin, hypovase, indoramin, doxazosin, cardura, doxadura, hydralazine, and minoxidil.
Use of antilipemic medication	Categorical (yes or not)	Field 20003, 6153, 6177	Self-reported use of lipid-lowering medications, including simvastatin, pravastatins, atrovastatins, fluvastatins, rosuvastatins, ezetimibe, nicotinic acid products, and fenofibrate.
Polygenic risk score of age-related macular degeneration(12)	Continuous	Field 26204	PRS scores were generated based on all UK biobank individuals and using a Bayesian approach applied to meta-analysed summary statistics obtained entirely from external GWAS data. A subsequent principal component-based ancestry centring step was applied to approximately centre the score distributions on zero across all ancestries, and the score distributions were also standardised. Please refer to the source literature for details.

References :

1.National Institute for Health and Care Excellence: Guidelines. Hypertension in adults: diagnosis and management. London: National Institute for Health and Care Excellence (NICE) Copyright © NICE 2022.; 2022.
2.Kopin L, Lowenstein C. Dyslipidemia. Ann Intern Med. 2017;167(11):Itc81-itc96. Epub 2017/12/06. doi: 10.7326/aitc201712050. PubMed PMID: 29204622.
3.Li FR, Hukportie DN, Yang J, Yang HH, Chen GC, Wu XB. Microvascular Burden and Incident Heart Failure Among Middle-Aged and Older Adults With Type 1 or Type 2 Diabetes. Diabetes Care. 2022. Epub 2022/08/10. doi: 10.2337/dc22-0177. PubMed PMID: 35944243.
4.Eastwood SV, Mathur R, Atkinson M, Brophy S, Sudlow C, Flaig R, et al. Algorithms for the Capture and Adjudication of Prevalent and Incident Diabetes in UK Biobank. PLoS One. 2016;11(9):e0162388. doi: 10.1371/journal.pone.0162388. PubMed PMID: 27631769.
5.Emanuelsson F, Marott S, Tybjærg-Hansen A, Nordestgaard BG, Benn M. Impact of Glucose Level on Micro- and Macrovascular Disease in the General Population: A Mendelian Randomization Study. Diabetes Care. 2020;43(4):894-902. Epub 2020/02/15. doi: 10.2337/dc19-1850. PubMed PMID: 32054721.
6.Nair ATN, Wesolowska-Andersen A, Brorsson C, Rajendrakumar AL, Hapca S, Gan S, et al. Heterogeneity in phenotype, disease progression and drug response in type 2 diabetes. Nat Med. 2022;28(5):982-8. Epub 2022/05/10. doi: 10.1038/s41591-022-01790-7. PubMed PMID: 35534565.
7.Vujkovic M, Keaton JM, Lynch JA, Miller DR, Zhou J, Tcheandjieu C, et al. Discovery of 318 new risk loci for type 2 diabetes and related vascular outcomes among 1.4 million participants in a multi-ancestry meta-analysis. Nat Genet. 2020;52(7):680-91. Epub 2020/06/17. doi: 10.1038/s41588-020-0637-y. PubMed PMID: 32541925; PubMed Central PMCID: PMC7343592.
8.Guggenheim JA, Williams C. Role of Educational Exposure in the Association Between Myopia and Birth Order. JAMA Ophthalmol. 2015;133(12):1408-14. Epub 2015/10/09. doi: 10.1001/jamaophthalmol.2015.3556. PubMed PMID: 26448589; PubMed Central PMCID: PMC4681114.
9.Guggenheim JA, Williams C. Childhood febrile illness and the risk of myopia in UK Biobank participants. Eye (Lond). 2016;30(4):608-14. Epub 2016/02/06. doi: 10.1038/eye.2016.7. PubMed PMID: 26846593; PubMed Central PMCID: PMC4834038.
10.Zhang YB, Chen C, Pan XF, Guo J, Li Y, Franco OH, et al. Associations of healthy lifestyle and socioeconomic status with mortality and incident cardiovascular disease: two prospective cohort studies. Bmj. 2021;373:n604. Epub 2021/04/16. doi: 10.1136/bmj.n604. PubMed PMID: 33853828; PubMed Central PMCID: PMC8044922.
11.Daviet R, Aydogan G, Jagannathan K, Spilka N, Koellinger PD, Kranzler HR, et al. Associations between alcohol consumption and gray and white matter volumes in the UK Biobank. Nat Commun. 2022;13(1):1175. Epub 2022/03/06. doi: 10.1038/s41467-022-28735-5. PubMed PMID: 35246521; PubMed Central PMCID: PMC8897479.

Table S2 Baseline characteristics of participants in the prospective cohort analysis stratified by tertiles of RST

Characteristics*	RST tertiles			Overall
	Low (0-3h/d)	Moderate (3.5-4h/d)	High (>4h/d)	
N	221730	104188	157021	482939
Age, years (mean (SD))	55.33 (8.12)	57.04 (7.95)	57.71 (7.90)	56.47 (8.09)
Sex, male (%)	93014 (41.9)	47800 (45.9)	80042 (51.0)	220856 (45.7)
Ethnicity, non-white (%)	12162 (5.5)	4640 (4.5)	9428 (6.0)	26230 (5.4)
Educational background, lower than college/university (%)	133617 (60.3)	73794 (70.8)	117235 (74.7)	324646 (67.2)
Townsend index (mean (SD))	-1.47 (2.98)	-1.51 (2.97)	-1.03 (3.23)	-1.33 (3.07)
Smoking status, (%)				
None	131441 (59.3)	56325 (54.1)	76746 (48.9)	264512 (54.8)
Former	70121 (31.6)	37367 (35.9)	60202 (38.3)	167690 (34.7)
Current	20168 (9.1)	10496 (10.1)	20073 (12.8)	50737 (10.5)
History of diabetes, yes (%)	9448 (4.3)	6019 (5.8)	13816 (8.8)	29283 (6.1)
History of cardiovascular diseases, yes (%)	8018 (3.6)	5400 (5.2)	11234 (7.2)	24652 (5.1)
History of hypertension, yes (%)	108024 (48.7)	59242 (56.9)	96496 (61.5)	263762 (54.6)
History of hyperlipidemia, yes (%)	111163 (50.1)	60810 (58.4)	99020 (63.1)	270993 (56.1)

* Quantitative data were expressed as (mean (SD)) and qualitative data were expressed as n (%).

RST: recreational screen time, SD: standard deviation.

Table S3 Baseline characteristics of participants in the prospective cohort analysis stratified according to the presence or absence of incident AMD

Characteristics*	Incident AMD		Overall
	No	Yes	
N	475443	7496	482939
Age, years (mean (SD))	56.37 (8.08)	62.81 (5.37)	56.47 (8.09)
Sex, male (%)	217896 (45.8)	2960 (39.5)	220856 (45.7)
Ethnicity, non-white (%)	25859 (5.4)	371 (4.9)	26230 (5.4)
Educational background, lower than college/university (%)	319051 (67.1)	5595 (74.6)	324646 (67.2)
Townsend index (mean (SD))	-1.33 (3.07)	-1.33 (3.06)	-1.33 (3.07)
Smoking status, (%)			
None	260839 (54.9)	3673 (49.0)	264512 (54.8)
Former	164539 (34.6)	3151 (42.0)	167690 (34.7)
Current	50065 (10.5)	672 (9.0)	50737 (10.5)
History of diabetes, yes (%)	28451 (6.0)	832 (11.1)	29283 (6.1)
History of cardiovascular diseases, yes (%)	24006 (5.0)	646 (8.6)	24652 (5.1)
History of hypertension, yes (%)	258663 (54.4)	5099 (68.0)	263762 (54.6)
History of hyperlipidemia, yes (%)	266029 (56.0)	4964 (66.2)	270993 (56.1)
Recreational screen time, hours per day (mean (SD))	3.87 (2.11)	4.13 (2.06)	3.87 (2.11)

* Quantitative data were expressed as (mean (SD)) and qualitative data were expressed as n (%).

AMD: age-related macular degeneration, SD: standard deviation.

Table S4 Prospective estimates of the association between quintile form of total daily RST and age-related macular degeneration

Exposure	Unadjusted model		Partially-adjusted model*		Fully-adjusted model†	
	HR (95%CI)	*P*	HR (95%CI)	*P*	HR (95%CI)	*P*
**Total daily RST**						
0-2h/d	1 [Reference]	-	1 [Reference]	-	1 [Reference]	-
2.5-3h/d	1.14 (1.06 - 1.23)	< 0.001	1.06 (0.99 - 1.15)	0.093	1.06 (0.99 - 1.14)	0.112
3.5-4h/d	1.35 (1.26 - 1.45)	< 0.001	1.10 (1.02 - 1.18)	0.009	1.08 (1.00 - 1.16)	0.038
4.5-5h/d	1.49 (1.38 - 1.61)	< 0.001	1.11 (1.03 - 1.20)	0.007	1.07 (0.99 - 1.16)	0.072
>5h/d	1.60 (1.49 - 1.72)	< 0.001	1.27 (1.18 - 1.36)	< 0.001	1.18 (1.09 - 1.27)	< 0.001

* Adjusted for age and sex.

† Adjusted for age, sex, race, education, Townsend deprivation index, smoking status, hypertension, hyperlipidemia, diabetes, and cardiovascular diseases.

RST: recreational screen time, HR: hazards ratio, CI: confidence interval.

Table S5 Sensitivity analysis excluding participants with an AMD diagnosis record within 2 years of the baseline assessment

Exposure	Unadjusted model		Partially-adjusted model*		Fully-adjusted model†	
	HR (95%CI)	*P*	HR (95%CI)	*P*	HR (95%CI)	*P*
**Total daily recreational screen time**						
Per SD increase	1.13 (1.11 - 1.16)	< 0.001	1.08 (1.05 - 1.11)	< 0.001	1.05 (1.02 - 1.07)	< 0.001
Tertiles						
Lowest (0-3h/d)	1 [Reference]	-	1 [Reference]	-	1 [Reference]	-
Moderate (3.5-4h/d)	1.26 (1.19 - 1.34)	< 0.001	1.07 (1.00 - 1.13)	0.044	1.05 (0.98 - 1.11)	0.153
High (>4h/d)	1.44 (1.36 - 1.51)	< 0.001	1.15 (1.09 - 1.21)	< 0.001	1.09 (1.03 - 1.15)	0.002

* Adjusted for age and sex.

† Adjusted for age, sex, race, education, Townsend deprivation index, smoking status, hypertension, hyperlipidemia, diabetes, and cardiovascular diseases.

AMD: age-related macular degeneration, HR: hazards ratio, CI: confidence interval, SD: standard deviation.

Table S6 Sensitivity analysis excluding participants with self-reported diagnoses of AMD

Exposure	Unadjusted model		Partially-adjusted model*		Fully-adjusted model†	
	HR (95%CI)	*P*	HR (95%CI)	*P*	HR (95%CI)	*P*
**Total daily recreational screen time**						
Per SD increase	1.14 (1.12 - 1.16)	< 0.001	1.09 (1.06 - 1.11)	< 0.001	1.05 (1.03 - 1.08)	< 0.001
Tertiles						
Lowest (0-3h/d)	1 [Reference]	-	1 [Reference]	-	1 [Reference]	-
Moderate (3.5-4h/d)	1.27 (1.20 - 1.35)	< 0.001	1.07 (1.01 - 1.14)	0.017	1.05 (0.99 - 1.12)	0.085
High (>4h/d)	1.46 (1.39 - 1.53)	< 0.001	1.16 (1.11 - 1.22)	< 0.001	1.10 (1.04 - 1.16)	< 0.001

* Adjusted for age and sex.

† Adjusted for age, sex, race, education, Townsend deprivation index, smoking status, hypertension, hyperlipidemia, diabetes, and cardiovascular diseases.

AMD: age-related macular degeneration, HR: hazards ratio, CI: confidence interval, SD: standard deviation.

Table S7 Sensitivity analysis with additional adjustment*

Exposure	HR (95%CI)	*P*
**Total daily recreational screen time**		
Per SD increase	1.03 (1.00 - 1.06)	0.025
Tertiles		
Lowest (0-3h/d)	1 [Reference]	-
Moderate (3.5-4h/d)	1.07 (1.00 - 1.14)	0.058
High (>4h/d)	1.08 (1.01 - 1.15)	0.017

* Adjusted for age, sex, race, education, Townsend deprivation index, smoking status, BMI, diet, alcohol consumption, physical activity, history of diabetes, hypertension, hyperlipidemia, and cardiovascular diseases, use of antidiabetic, antihypertensive, and antilipemic medication, and PRS of AMD.

BMI: body mass index, MET: metabolic equivalent task, PRS: polygenic risk score, AMD: age-related macular degeneration, SD: standard deviation, HR: hazards ratio, CI: confidence interval.

Table S8 Subgroup and interaction analysis for prospective estimates of the association between RST and AMD

Subgroup*	Count	Percent	HR (95% CI)	P value	P for interaction
**Age**					
<=60 years old	223661	57.8	1.05 (0.99 - 1.10)	0.083	0.947
>60 years old	162982	42.2	1.05 (1.02 - 1.08)	0.001	
**Gender**					
Female	201793	52.2	1.04 (1.00 - 1.08)	0.042	0.649
Male	184850	47.8	1.04 (1.00 - 1.09)	0.030	
**Employment status**					
Looking after family	11296	2.9	0.98 (0.80 - 1.20)	0.858	0.690
Retired/not working	135992	35.2	1.04 (1.01 - 1.08)	0.018	
Working/student	239355	61.9	1.03 (0.98 - 1.07)	0.274	
**Obesity status**					
Normal/overweight (BMI<=30kg/m2)	296477	76.7	1.04 (1.00 - 1.07)	0.035	0.652
Obesity (BMI>30kg/m2)	90166	23.3	1.04 (1.00 - 1.07)	0.014	
**Smoking status**					
Never	211682	54.7	1.04 (1.00 - 1.08)	0.062	0.980
Former/current	174961	45.3	1.04 (1.01 - 1.08)	0.020	

* Adjusted for age, sex, race, education, Townsend deprivation index, BMI, smoking status, hypertension, hyperlipidemia, diabetes, and cardiovascular diseases when these were not the subgroup used; HR corresponds to RST per SD change.

RST: recreational screen time, AMD: age-related macular degneration, BMI: body mass index, SD: standard deviation, HR: hazards ratio, CI: confidence interval.

Table S9 Information on GWAS datasets in the MR study

Phenotype	Type	Sample size (overall or cases/controls)	Ancestry	Consortium	Published year	Source	URL
Self-reported recreational screen time (1)	Continuous (SD)	526,725	European	meta-analysis	2022	PMID: 36071172	https://www.ebi.ac.uk/gwas/downloads/summary-statistics
Age-related macular degeneration (2)	Binary	8,913/348,936	European	FinnGen	2023	FREEZE 9	https://r9.finngen.fi/
Body mass index (3)	Continuous (SD)	806,834	European	GIANT	2019	PMID: 30239722	https://portals.broadinstitute.org/collaboration/giant/index.php/GIANT_consortium_data_files
Glycated hemoglobin (4)	Categorical (quantile)	361,194	European	UK Biobank	2018	Neale Lab (Pan UKBB)	https://pan.ukbb.broadinstitute.org/downloads
Lifetime smoking index (5)	Continuous (SD)	462,690	European	UK Biobank	2020	PMID: 31689377	https://doi.org/10.5523/bris.10i96zb8gm0j81yz0q6ztei23d
High-density lipoprotein cholesterol (6)	Continuous (SD)	1,320,016	European	GLGC	2021	PMID: 34887591	https://csg.sph.umich.edu/willer/public/glgc-lipids2021
Inner segment layer of photoreceptor cells (7)	Continuous (SD)	31,135	European	UK Biobank	2023	PMID: 36848389	https://www.ebi.ac.uk/gwas/downloads/summary-statistics
Outer segment layer of photoreceptor cells (7)	Continuous (SD)	31,135	European	UK Biobank	2023	PMID: 36848389	https://www.ebi.ac.uk/gwas/downloads/summary-statistics
Ganglion cell-inner plexiform layer (8)	Continuous (SD)	31,434	European	UK Biobank	2021	PMID: 33979322	https://www.ebi.ac.uk/gwas/downloads/summary-statistics
Retinal nerve fiber layer (8)	Continuous (SD)	31,434	European	UK Biobank	2021	PMID: 33979322	https://www.ebi.ac.uk/gwas/downloads/summary-statistics

GWAS: genome-wide association study, MR: Mendelian randomization, URL: Uniform Resource Locator, FREEZE 9: the 9th edition of GWAS summary level data of FinnGen study, SD: standard deviation, GIANT: the Genetic Investigation of ANthropometric Traits consortium, GLGC: the Global Lipids Genetics Consortium.

References：

1.Wang Z, Emmerich A, Pillon NJ, Moore T, Hemerich D, Cornelis MC, et al. Genome-wide association analyses of physical activity and sedentary behavior provide insights into underlying mechanisms and roles in disease prevention. Nat Genet. 2022;54(9):1332-44.
2.Kurki MI, Karjalainen J, Palta P, Sipilä TP, Kristiansson K, Donner KM, et al. FinnGen provides genetic insights from a well-phenotyped isolated population. Nature. 2023;613(7944):508-18.
3.Pulit SL, Stoneman C, Morris AP, Wood AR, Glastonbury CA, Tyrrell J, et al. Meta-analysis of genome-wide association studies for body fat distribution in 694 649 individuals of European ancestry. Hum Mol Genet. 2019;28(1):166-74.
4.Pan-UKB team. 2020. Available from: https://pan.ukbb.broadinstitute.org.
5.Wootton RE, Richmond RC, Stuijfzand BG, Lawn RB, Sallis HM, Taylor GMJ, et al. Evidence for causal effects of lifetime smoking on risk for depression and schizophrenia: a Mendelian randomisation study. Psychol Med. 2020;50(14):2435-43.
6.Graham SE, Clarke SL, Wu KH, Kanoni S, Zajac GJM, Ramdas S, et al. The power of genetic diversity in genome-wide association studies of lipids. Nature. 2021;600(7890):675-9.
7.Currant H, Fitzgerald TW, Patel PJ, Khawaja AP, Webster AR, Mahroo OA, et al. Sub-cellular level resolution of common genetic variation in the photoreceptor layer identifies continuum between rare disease and common variation. PLoS Genet. 2023;19(2):e1010587.
8.Currant H, Hysi P, Fitzgerald TW, Gharahkhani P, Bonnemaijer PWM, Senabouth A, et al. Genetic variation affects morphological retinal phenotypes extracted from UK Biobank optical coherence tomography images. PLoS Genet. 2021;17(5):e1009497.

Table S10 Forward UVMR estimating the association of RST and AMD

Exposure	Outcome	Method	No. of SNPs	β	S.E.	OR	Lower 95%CI	Upper 95%CI	*P*	Directional pleiotropy test		Heterogeneity test		MR-Steiger directionality test
										MR-Egger intercept	*P*	Q	*P*	
RST	AMD	Inverse variance weighted	49	0.235	0.116	1.26	1.01	1.59	0.042	-0.00900	0.503	63.602	0.065	Correct causal direction
		MR Egger	49	0.557	0.492	1.75	0.67	4.58	0.263			62.992	0.059	
		Weighted median	49	0.119	0.154	1.13	0.83	1.52	0.437			-	-	
		MR PRESSO	0*	0.235	0.116	1.26	1.01	1.59	0.048			-	-	

* NO. of outliers.

UVMR: univariable Mendelian randomization, RST: recreational screen time, AMD: age-related macular degeneration, PRESSO: pleiotropy residual sum and outlier, SNP: single nucleotide polymorphism, S.E.: standard error, OR: odds ratio, CI: confidence interval.

Table S11 List of 49 conditionally independent genetic variants used as instruments of RST in the UVMR analysis

SNPs	Chr	Position	Effect Allele	Other Allele	EAF	Closest Gene	GWAS for RST (UK Biobank-Wang et.al)			GWAS for AMD (FinnGen-FREEZE 9)			R square	F statistics
							β	SE	*P*	β	SE	*P*		
rs10041724	5	124273520	T	C	0.807	ZNF608	0.025	0.004	1.51E-08	0.019	0.021	3.54E-01	6.08E-05	32.025
rs10059100	5	120100784	A	G	0.607	PRR16	-0.022	0.004	4.68E-08	0.028	0.016	8.51E-02	5.77E-05	30.391
rs10400776	14	97326366	A	C	0.259	VRK1	-0.026	0.004	3.45E-09	0.024	0.019	2.08E-01	6.58E-05	34.649
rs10772643	12	13415288	T	C	0.892	EMP1	-0.039	0.006	5.88E-10	0.019	0.022	3.71E-01	7.32E-05	38.560
rs10889193	1	61106174	A	C	0.555	NFIA	0.024	0.004	4.94E-10	0.002	0.016	8.79E-01	7.37E-05	38.822
rs11074658	16	10308335	T	C	0.591	GRIN2A	-0.024	0.004	9.21E-10	-0.002	0.017	9.00E-01	7.13E-05	37.555
rs114590429	2	166176789	A	C	0.116	SCN2A	0.038	0.006	3.03E-10	0.019	0.021	3.71E-01	7.46E-05	39.271
rs11587591	1	209762875	A	G	0.734	CAMK1G	0.022	0.004	2.23E-08	-0.011	0.019	5.49E-01	5.99E-05	31.532
rs1188887	6	139257866	T	C	0.667	REPS1	0.026	0.004	2.47E-10	0.013	0.017	4.24E-01	7.52E-05	39.598
rs11972285	7	99025591	A	G	0.846	ATP5J2-PTCD1,PTCD1	0.038	0.006	5.13E-12	0.002	0.019	9.02E-01	8.93E-05	47.020
rs12206846	6	108238917	A	G	0.405	SEC63	0.020	0.004	2.10E-08	0.026	0.016	1.09E-01	5.80E-05	30.556
rs12214364	6	67556372	T	G	0.587	EYS	-0.020	0.004	4.87E-08	0.015	0.017	3.65E-01	5.68E-05	29.945
rs12463321	19	37651855	A	G	0.148	CTC-454I21.3,ZNF585A	-0.032	0.006	6.99E-09	-0.037	0.022	9.08E-02	6.31E-05	33.219
rs12678836	8	92690148	A	C	0.424	RUNX1T1	0.023	0.004	5.37E-11	0.001	0.016	9.53E-01	8.34E-05	43.938
rs13017586	2	147847198	A	G	0.849	ACVR2A	-0.040	0.005	8.25E-14	0.026	0.021	2.25E-01	1.05E-04	55.144
rs13089152	3	84765574	T	C	0.669	CADM2	-0.023	0.004	1.53E-08	-0.032	0.017	6.83E-02	6.18E-05	32.573
rs1391954	11	88575965	T	G	0.444	GRM5	0.025	0.004	1.51E-09	-0.014	0.016	3.83E-01	7.12E-05	37.515
rs17621391	7	140176596	T	C	0.735	MKRN1	0.024	0.004	2.11E-09	0.023	0.019	2.21E-01	6.67E-05	35.127
rs1860337	17	60851559	T	C	0.595	10-Mar	-0.025	0.004	9.08E-11	-0.013	0.016	4.24E-01	7.99E-05	42.083
rs1947066	5	161101615	A	G	0.803	GABRA6	0.030	0.004	8.54E-12	0.016	0.021	4.57E-01	8.71E-05	45.870
rs1999065	9	120514574	T	C	0.338	TLR4	0.025	0.004	1.24E-11	0.023	0.016	1.67E-01	8.60E-05	45.289
rs249960	5	96164771	A	G	0.818	ERAP1	0.030	0.005	2.43E-09	-0.018	0.021	3.97E-01	6.74E-05	35.521
rs2738284	2	217311609	A	G	0.329	SMARCAL1	-0.022	0.004	7.05E-09	0.015	0.016	3.74E-01	6.41E-05	33.765
rs2964252	5	152067929	A	G	0.316	NMUR2	-0.024	0.004	3.16E-10	-0.011	0.017	5.30E-01	7.66E-05	40.340
rs34864022	9	22609110	A	G	0.934	DMRTA1	-0.048	0.008	4.71E-10	-0.051	0.034	1.40E-01	7.28E-05	38.345
rs3759344	12	6862646	A	G	0.106	MLF2	0.045	0.006	4.26E-13	0.030	0.030	3.03E-01	1.01E-04	53.149
rs421151	8	73462574	A	G	0.916	KCNB2	-0.036	0.006	1.09E-08	0.020	0.027	4.55E-01	6.20E-05	32.653
rs4416502	4	77030872	A	G	0.200	ART3	0.029	0.005	1.38E-09	0.006	0.019	7.36E-01	6.98E-05	36.754
rs4460001	4	130275243	A	C	0.432	C4orf33	-0.019	0.004	4.45E-08	-0.007	0.016	6.65E-01	5.77E-05	30.407
rs4483592	11	65990439	T	C	0.163	PACS1	0.036	0.005	3.97E-12	0.042	0.021	4.31E-02	9.10E-05	47.929
rs558134	6	12693454	T	C	0.384	PHACTR1	-0.023	0.004	5.05E-10	-0.041	0.016	1.09E-02	7.42E-05	39.062
rs57092155	7	53856368	T	C	0.783	POM121L12	-0.027	0.005	8.80E-09	0.003	0.019	8.74E-01	6.17E-05	32.514
rs58087899	1	1863026	A	G	0.456	CFAP74	-0.022	0.004	4.35E-08	0.030	0.016	6.44E-02	5.85E-05	30.802
rs6010651	20	62418243	A	C	0.620	ZBTB46	0.024	0.004	3.34E-09	0.002	0.017	9.09E-01	6.55E-05	34.515
rs6102913	20	41202958	T	C	0.474	PTPRT	-0.019	0.004	2.97E-08	0.004	0.016	8.21E-01	5.77E-05	30.407
rs62134209	2	45093457	A	G	0.953	SIX3	0.054	0.009	3.28E-09	0.033	0.038	3.84E-01	6.64E-05	34.953
rs6556840	5	93463902	A	G	0.337	FAM172A	0.020	0.004	4.32E-08	-0.017	0.016	2.99E-01	5.77E-05	30.399
rs657412	13	99047250	T	G	0.105	FARP1	-0.033	0.006	1.40E-08	-0.023	0.039	5.61E-01	6.15E-05	32.372
rs6674314	1	243920895	A	G	0.813	AKT3	0.027	0.005	2.30E-08	-0.021	0.019	2.62E-01	5.94E-05	31.269
rs6727997	2	146346285	A	G	0.345	ZEB2	-0.021	0.004	2.04E-08	-0.055	0.018	1.95E-03	6.00E-05	31.603
rs68049022	10	66407019	T	C	0.798	REEP3	0.031	0.005	6.18E-11	0.031	0.022	1.63E-01	8.12E-05	42.793
rs72671494	8	93195457	T	C	0.860	RUNX1T1	-0.035	0.006	5.72E-10	0.009	0.025	7.09E-01	7.37E-05	38.839
rs73405293	12	117522917	A	G	0.147	TESC	-0.031	0.005	1.35E-08	0.000	0.022	9.92E-01	6.18E-05	32.532
rs7430216	3	75201030	T	C	0.223	FRG2C	0.025	0.004	2.50E-09	0.032	0.019	9.27E-02	6.73E-05	35.431
rs7616518	3	83530809	A	G	0.450	CADM2	0.020	0.004	2.60E-08	0.000	0.016	9.90E-01	5.95E-05	31.360
rs78394231	6	107649123	T	C	0.902	PDSS2	-0.038	0.007	3.53E-09	-0.055	0.026	3.31E-02	6.52E-05	34.358
rs78451709	18	41515058	T	C	0.795	SYT4	-0.028	0.005	9.66E-09	0.016	0.021	4.53E-01	6.32E-05	33.302
rs7875078	9	14494845	A	C	0.452	NFIB	0.020	0.004	7.41E-09	0.007	0.016	6.71E-01	6.45E-05	33.972
rs9867121	3	114631548	A	C	0.183	ZBTB20	-0.032	0.005	2.02E-10	-0.024	0.021	2.59E-01	7.63E-05	40.195

RST: recreational screen time, UVMR: univariable Mendelian randomization, SNPs: single nucleotide polymorphisms, Chr: chromosome, EAF: effect allele frequency, GWAS: genome-wide association study, SE: standard error, AMD: age-related macular degeneration.

Table S12 Reverse UVMR estimating the association of RST and AMD

Exposure	Outcome	Method	No. of SNPs	β	S.E.	Lower 95%CI	Upper 95%CI	*P*
AMD	RST	Inverse variance weighted	21	0.001	0.007	-0.013	0.015	0.913
		MR Egger	21	-0.004	0.013	-0.030	0.022	0.766
		Weighted median	21	-0.010	0.007	-0.024	0.005	0.192
		MR PRESSO	0*	0.001	0.007	-0.013	0.015	0.914

* NO. of outliers.

UVMR: univariable Mendelian randomization, RST: recreational screen time, AMD:age-relatd macular degeneration, PRESSO: pleiotropy residual sum and outlier, SNP: single nucleotide polymorphism, CI: confidence interval, S.E.: standard error.

Table S13 UVMR estimating the association of RST and AMD using instrumental variables with relaxing linkage disequilibrium threshold

Exposure	Outcome	Method	No. of SNPs	β	S.E.	OR	Lower 95%CI	Upper 95%CI	*P*	Directional pleiotropy test		Heterogeneity test		MR-Steiger directionality test
										MR-Egger intercept	*P*	Q	*P*	
RST (r2 < 0.01 within a 5000 kb window)	AMD	Inverse variance weighted	54	0.260	0.101	1.30	1.07	1.58	0.010	-0.01300	0.239	58.193	0.290	Correct causal direction
		MR Egger	54	0.743	0.418	2.10	0.93	4.77	0.081			56.647	0.306	
		Weighted median	54	0.207	0.136	1.23	0.94	1.61	0.129			-	-	
		MR PRESSO	0*	0.260	0.101	1.30	1.07	1.58	0.012			-	-	

* NO. of outliers.

UVMR: univariable Mendelian randomization, RST: recreational screen time, AMD:age-relatd macular degeneration, PRESSO: pleiotropy residual sum and outlier, SNP: single nucleotide polymorphism, S.E.: standard error, OR: odds ratio, CI: confidence interval.

Table S14 UVMR estimating the association of RST and AMD using instrumental variables located in the KLC2 gene region

Exposure	Outcome	Method	No. of SNPs	β	S.E.	OR	Lower 95%CI	Upper 95%CI	*P*	Directional pleiotropy test		Heterogeneity test		MR-Steiger directionality test
										MR-Egger intercept	*P*	Q	*P*	
RST (KLC2)	AMD	Inverse variance weighted	6	1.310	0.428	3.70	1.60	8.57	0.002	-0.02900	0.408	3.361	0.645	Correct causal direction
		MR Egger	6	2.376	1.231	10.76	0.96	120.24	0.126			2.507	0.643	
		Weighted median	6	1.248	0.501	3.49	1.31	9.30	0.013			-	-	
		MR PRESSO	0*	1.310	0.351	3.70	1.86	7.37	0.014			-	-	

* NO. of outliers.

UVMR: univariable Mendelian randomization, RST: recreational screen time, AMD:age-relatd macular degeneration, PRESSO: pleiotropy residual sum and outlier, SNP: single nucleotide polymorphism, S.E.: standard error, OR: odds ratio, CI: confidence interval.

Table S15 Colocalization analysis of recreational screen time and AMD in the KLC2 gene region

Exposure	Outcome	No. of SNPs	PP.H0	PP.H1	PP.H2	PP.H3	PP.H4
Recreational screen time	AMD	1033	6.55E-07	0.862	5.46E-08	0.072	0.067

AMD:age-relatd macular degeneration, SNP: single nucleotide polymorphism.

Table S16 MVMR estimating the association of RST and AMD with adjustment for potential confounders

Method	N.SNPs	Exposure	OR	Lower 95%CI	Upper 95%CI	S.E.	*P*	MVMR directional pleiotropy test	
								Egger intercept	*P*
**Models with mutual adjustment for RST and BMI**									
MV-IVW	460	RST	0.89	0.71	1.12	0.118	0.323	-	-
		BMI	1.19	1.03	1.37	0.074	0.020		
MVMR Egger		RST	0.90	0.71	1.13	0.119	0.365	-0.0015	0.541
		BMI	1.29	0.95	1.77	0.159	0.107		
**Models with mutual adjustment for RST and HbA1c**									
MV-IVW	313	RST	1.58	1.07	2.31	0.196	0.020	-	-
		HbA1c	1.00	0.92	1.09	0.043	0.962		
MVMR Egger		RST	1.56	1.06	2.29	0.198	0.026	0.0012	0.628
		HbA1c	0.97	0.83	1.13	0.078	0.705		
**Models with mutual adjustment for RST and LSI**									
MV-IVW	110	RST	0.86	0.53	1.42	0.252	0.564	-	-
		LSI	1.48	0.87	2.53	0.273	0.150		
MVMR Egger		RST	0.91	0.54	1.53	0.267	0.712	0.0083	0.575
		LSI	2.21	0.49	9.89	0.765	0.300		
**Models with mutual adjustment for RST and HDLC**									
MV-IVW	389	RST	1.41	1.04	1.92	0.156	0.026	-	-
		HDLC	1.25	1.13	1.40	0.054	0.000		
MVMR Egger		RST	1.38	1.00	1.90	0.163	0.050	0.0019	0.583
		HDLC	1.29	1.11	1.51	0.079	0.001		

MVMR: multivariable Mendelian randomization, RST: recreational screen time, AMD: age-related macular degeneration, N.SNP: number of single nucleotide polymorphisms included in the model, OR: odds ratio, CI: confidence interval, S.E.: standard error, MV-IVW: multivariable inverse variance weighted, BMI: body mass index, HbA1c: glycated hemoglobin, LSI: lifetime smoking index, HDLC: high-density lipoprotein cholesterol.

Table S17 General characteristics of participants in the cross-sectional analysis stratified by tertiles of RST

Characteristics*	RST tertiles			Overall
	Low (0-3h/d)	Moderate (3.5-4h/d)	High (>4h/d)	
N	17364	8319	12795	38478
Age, years (mean (SD))	54.45 (8.11)	56.02 (8.10)	56.74 (8.09)	55.55 (8.17)
Sex, male (%)	7317 (42.1)	3788 (45.5)	6452 (50.4)	17557 (45.6)
Ethnicity, non-white (%)	1327 (7.6)	565 (6.8)	1218 (9.5)	3110 (8.1)
Educational background, lower than college/university (%)	9822 (56.6)	5561 (66.8)	9005 (70.4)	24388 (63.4)
Townsend index (mean (SD))	-1.15 (2.88)	-1.30 (2.88)	-0.93 (3.04)	-1.11 (2.94)
Smoking status, (%)				
None	10302 (59.5)	4598 (55.5)	6477 (50.8)	21377 (55.7)
Former	5550 (32.0)	2888 (34.8)	4782 (37.5)	13220 (34.5)
Current	1472 (8.5)	802 (9.7)	1499 (11.7)	3773 (9.8)
History of cardiovascular diseases, yes (%)	510 (2.9)	274 (3.3)	669 (5.2)	1453 (3.8)
History of hypertension, yes (%)	7457 (42.9)	4238 (50.9)	7072 (55.3)	18767 (48.8)
History of hyperlipidemia, yes (%)	7980 (46.0)	4412 (53.0)	7428 (58.1)	19820 (51.5)
Visual acuity of the better eye, logMAR (mean (SD))	-0.09 (0.10)	-0.08 (0.10)	-0.08 (0.10)	-0.08 (0.10)
Mean intraocular pressure, mmHg (mean (SD))	15.20 (2.75)	15.36 (2.75)	15.38 (2.77)	15.30 (2.76)
Mean spherical equivalent, diopters (mean (SD))	-0.15 (1.95)	-0.01 (1.92)	0.11 (1.92)	-0.03 (1.94)
RNFL, microns (mean (SD))	28.67 (4.02)	28.53 (4.11)	28.26 (4.06)	28.50 (4.06)
GC-IPL, microns (mean (SD))	75.02 (5.39)	74.80 (5.49)	74.54 (5.52)	74.82 (5.46)
INL, microns (mean (SD))	32.62 (2.26)	32.65 (2.27)	32.63 (2.26)	32.63 (2.26)
INL-ELM, microns (mean (SD))	80.75 (6.23)	80.65 (6.18)	80.50 (6.24)	80.65 (6.22)
ELM-ISOS, microns (mean (SD))	23.71 (1.51)	23.59 (1.50)	23.46 (1.44)	23.60 (1.49)
ISOS-RPE, microns (mean (SD))	38.25 (3.97)	38.09 (3.85)	38.01 (3.82)	38.13 (3.90)
INL-RPE, microns (mean (SD))	142.71 (7.62)	142.33 (7.48)	141.97 (7.56)	142.38 (7.58)
RPE, microns (mean (SD))	25.31 (2.80)	25.26 (2.74)	25.25 (2.76)	25.28 (2.77)
Macula, microns (mean (SD))	279.04 (12.98)	278.33 (12.92)	277.42 (13.16)	278.35 (13.05)

* Quantitative data were expressed as (mean (SD)) and qualitative data were expressed as n (%).

RST: recreational screen time, SD: standard deviation, RNFL: average retinal nerve fiber layer thickness across all subfields; GC-IPL: average ganglion cell-inner plexiform layer thickness across all subfields; INL: average inner nuclear layer thickness across all subfields; INL-ELM: average thickness measured between the inner nuclear layer to the external limiting membrane across all subfields, mainly reflecting photoreceptor cell axons, synaptic terminals, and nuclei; ELM-ISOS: average thickness measured between the external limiting membrane to the junction between the inner and outer photoreceptor segments across all subfields, mainly reflecting the inner segment of photoreceptor cells; ISOS-RPE: average thickness measured between the junction between the inner and outer photoreceptor segments to the retinal pigment epithelium across all subfields, mainly reflecting the outer segment of photoreceptor cells; INL-RPE: average thickness measured between the inner nuclear layer to the retinal pigment epithelium across all subfields, mainly reflecting the total photoreceptor layer thickness; RPE: average retinal pigment epithelium thickness across all subfields; Macula: average macular thickness measured from the inner limiting membrane to the retinal pigment epithelium across all subfields.

Table S18 Cross-sectional estimates of the association between daily recreational computer using time and retinal thickness

Outcome*	Unadjusted model		Partially-adjusted model†		Fully-adjusted model‡	
	β (95%CI)	*P*	β (95%CI)	*P*	β (95%CI)	*P*
RNFL	0.03 (-0.01 - 0.07)	0.144	0.03 (-0.01 - 0.07)	0.114	0.02 (-0.02 - 0.06)	0.333
GC-IPL	0.01 (-0.04 - 0.06)	0.601	-0.07 (-0.12 - -0.01)	0.012	-0.05 (-0.10 - 0.00)	0.076
INL	-0.02 (-0.04 - 0.00)	0.087	-0.06 (-0.08 - -0.04)	< 0.001	-0.03 (-0.05 - -0.01)	0.007
INL-ELM	-0.01 (-0.06 - 0.05)	0.825	-0.07 (-0.13 - -0.01)	0.025	-0.01 (-0.07 - 0.05)	0.750
ELM-ISOS	0.04 (0.03 - 0.06)	< 0.001	0.01 (-0.01 - 0.02)	0.281	0.00 (-0.02 - 0.01)	0.570
ISOS-RPE	0.05 (0.02 - 0.09)	0.004	0.05 (0.02 - 0.09)	0.005	0.05 (0.01 - 0.09)	0.006
INL-RPE	0.09 (0.02 - 0.16)	0.011	-0.01 (-0.08 - 0.06)	0.850	0.04 (-0.03 - 0.11)	0.286
RPE	0.02 (-0.01 - 0.05)	0.141	-0.05 (-0.07 - -0.02)	< 0.001	-0.05 (-0.07 - -0.02)	< 0.001
Macula	0.12 (-0.01 - 0.24)	0.064	-0.10 (-0.22 - 0.02)	0.108	-0.02 (-0.14 - 0.11)	0.791

* Effect of per SD increase of recreational computer using time on the outcomes.

† Adjusted for age and sex.

‡ Adjusted for age, sex, race, education, Townsend deprivation index, smoking status, history of hypertension, hyperlipidemia and cardiovascular diseases, visual acuity of the better eye, mean intraocular pressure and mean spherical equivalent of both eyes.

CI: confidence interval, RNFL: average retinal nerve fiber layer thickness across all subfields; GC-IPL: average ganglion cell-inner plexiform layer thickness across all subfields; INL: average inner nuclear layer thickness across all subfields; INL-ELM: average thickness measured between the inner nuclear layer to the external limiting membrane across all subfields, mainly reflecting photoreceptor cell axons, synaptic terminals, and nuclei; ELM-ISOS: average thickness measured between the external limiting membrane to the junction between the inner and outer photoreceptor segments across all subfields, mainly reflecting the inner segment of photoreceptor cells; ISOS-RPE: average thickness measured between the junction between the inner and outer photoreceptor segments to the retinal pigment epithelium across all subfields, mainly reflecting the outer segment of photoreceptor cells; INL-RPE: average thickness measured between the inner nuclear layer to the retinal pigment epithelium across all subfields, mainly reflecting the total photoreceptor layer thickness; RPE: average retinal pigment epithelium thickness across all subfields; Macula: average macular thickness measured from the inner limiting membrane to the retinal pigment epithelium across all subfields.

Table S19 Cross-sectional estimates of the association between daily TV watching time and retinal thickness

Outcome*	Unadjusted model		Partially-adjusted model†		Fully-adjusted model‡	
	β (95%CI)	*P*	β (95%CI)	*P*	β (95%CI)	*P*
RNFL	-0.27 (-0.31 - -0.23)	< 0.001	-0.19 (-0.23 - -0.15)	< 0.001	-0.08 (-0.12 - -0.03)	0.001
GC-IPL	-0.28 (-0.34 - -0.22)	< 0.001	-0.10 (-0.16 - -0.05)	< 0.001	-0.17 (-0.23 - -0.11)	< 0.001
INL	0.03 (0.01 - 0.05)	0.015	0.06 (0.04 - 0.08)	< 0.001	-0.01 (-0.03 - 0.01)	0.404
INL-ELM	-0.13 (-0.20 - -0.07)	< 0.001	-0.18 (-0.25 - -0.11)	< 0.001	-0.23 (-0.30 - -0.16)	< 0.001
ELM-ISOS	-0.17 (-0.18 - -0.15)	< 0.001	-0.12 (-0.13 - -0.10)	< 0.001	-0.07 (-0.09 - -0.06)	< 0.001
ISOS-RPE	-0.21 (-0.25 - -0.17)	< 0.001	-0.24 (-0.28 - -0.20)	< 0.001	-0.17 (-0.22 - -0.13)	< 0.001
INL-RPE	-0.52 (-0.59 - -0.44)	< 0.001	-0.54 (-0.62 - -0.46)	< 0.001	-0.47 (-0.56 - -0.39)	< 0.001
RPE	-0.04 (-0.07 - -0.01)	0.005	0.04 (0.01 - 0.07)	0.015	-0.02 (-0.05 - 0.01)	0.152
Macula	-1.04 (-1.17 - -0.90)	< 0.001	-0.77 (-0.90 - -0.63)	< 0.001	-0.74 (-0.88 - -0.59)	< 0.001

* Effect of per SD increase of TV watching time on the outcomes.

† Adjusted for age and sex.

‡ Adjusted for age, sex, race, education, Townsend deprivation index, smoking status, history of hypertension, hyperlipidemia and cardiovascular diseases, visual acuity of the better eye, mean intraocular pressure and mean spherical equivalent of both eyes.

CI: confidence interval, RNFL: average retinal nerve fiber layer thickness across all subfields; GC-IPL: average ganglion cell-inner plexiform layer thickness across all subfields; INL: average inner nuclear layer thickness across all subfields; INL-ELM: average thickness measured between the inner nuclear layer to the external limiting membrane across all subfields, mainly reflecting photoreceptor cell axons, synaptic terminals, and nuclei; ELM-ISOS: average thickness measured between the external limiting membrane to the junction between the inner and outer photoreceptor segments across all subfields, mainly reflecting the inner segment of photoreceptor cells; ISOS-RPE: average thickness measured between the junction between the inner and outer photoreceptor segments to the retinal pigment epithelium across all subfields, mainly reflecting the outer segment of photoreceptor cells; INL-RPE: average thickness measured between the inner nuclear layer to the retinal pigment epithelium across all subfields, mainly reflecting the total photoreceptor layer thickness; RPE: average retinal pigment epithelium thickness across all subfields; Macula: average macular thickness measured from the inner limiting membrane to the retinal pigment epithelium across all subfields.

Table S20 Forward UVMR estimating the association of recreational screen time and retinal thickness

Exposure	Outcome	Method	No. of SNPs	β	S.E.	Lower 95%CI	Upper 95%CI	*P*	Directional pleiotropy test		Heterogeneity test		MR-Steiger directionality test
									MR-Egger intercept	*P*	Q	*P*	
RST	RNFL	Inverse variance weighted	55	-0.517	0.218	-0.943	-0.090	0.018	-0.01518	0.536	54.491	0.456	Correct causal direction
		MR Egger	55	0.035	0.912	-1.753	1.822	0.970			54.095	0.432	
		Weighted median	55	-0.746	0.322	-1.376	-0.115	0.020			-	-	
		MR PRESSO	0*	-0.517	0.218	-0.943	-0.090	0.021			-	-	
RST	GCIPL	Inverse variance weighted	55	-0.742	0.341	-1.411	-0.073	0.030	-0.04227	0.270	77.645	0.019	Correct causal direction
		MR Egger	55	0.794	1.419	-1.987	3.575	0.578			75.866	0.021	
		Weighted median	55	-0.945	0.413	-1.755	-0.135	0.022			-	-	
		MR PRESSO	0*	-0.742	0.341	-1.411	-0.073	0.034			-	-	
RST	IS	Inverse variance weighted	56	-0.064	0.079	-0.218	0.090	0.416	-0.00022	0.980	70.674	0.076	Correct causal direction
		MR Egger	56	-0.056	0.335	-0.712	0.600	0.868			70.673	0.063	
		Weighted median	56	-0.014	0.097	-0.205	0.176	0.883			-	-	
		MR PRESSO	0*	-0.064	0.079	-0.218	0.090	0.420			-	-	
RST	OS	Inverse variance weighted	55	-0.073	0.182	-0.429	0.284	0.690	0.02852	0.165	43.084	0.857	Correct causal direction
		MR Egger	55	-1.109	0.759	-2.596	0.378	0.150			41.104	0.883	
		Weighted median	55	0.066	0.264	-0.451	0.582	0.803			-	-	
		MR PRESSO	0*	-0.073	0.163	-0.391	0.246	0.657			-	-	

* NO. of outliers.

UVMR: univariable Mendelian randomization, RST: recreational screen time, RNFL: retinal nerve fiber layer, GCIPL: ganglion cell-inner plexiform layer, IS:inner segment layer of photoreceptor cells, OS:outer segment layer of photoreceptor cells, PRESSO: pleiotropy residual sum and outlier, SNP: single nucleotide polymorphism, S.E.: standard error, CI: confidence interval.

Table S21 Reverse UVMR estimating the association of recreational screen time and retinal thickness

Exposure	Outcome	Method	No. of SNPs	β	S.E.	Lower 95%CI	Upper 95%CI	*P*
RNFL	RST	Inverse variance weighted	23	0.002	0.004	-0.006	0.011	0.628
		MR Egger	23	-0.008	0.013	-0.034	0.018	0.564
		Weighted median	23	0.007	0.005	-0.002	0.016	0.138
		MR PRESSO	1*	0.006	0.004	-0.003	0.014	0.191
GCIPL	RST	Inverse variance weighted	17	0.003	0.003	-0.002	0.008	0.254
		MR Egger	17	0.000	0.007	-0.014	0.015	0.949
		Weighted median	17	0.004	0.004	-0.003	0.011	0.309
		MR PRESSO	0*	0.003	0.003	-0.002	0.008	0.271
IS	RST	Inverse variance weighted	13	-0.009	0.019	-0.045	0.027	0.630
		MR Egger	13	-0.010	0.063	-0.133	0.114	0.882
		Weighted median	13	-0.018	0.019	-0.056	0.019	0.333
		MR PRESSO	0*	-0.009	0.019	-0.045	0.027	0.638
OS	RST	Inverse variance weighted	30	0.000	0.003	-0.007	0.006	0.919
		MR Egger	30	0.016	0.009	-0.003	0.035	0.101
		Weighted median	30	-0.003	0.004	-0.011	0.005	0.496
		MR PRESSO	0*	0.000	0.003	-0.007	0.006	0.919

* NO. of outliers.

UVMR: univariable Mendelian randomization, RNFL: retinal nerve fiber layer, GCIPL: ganglion cell-inner plexiform layer, IS:inner segment layer of photoreceptor cells, OS:outer segment layer of photoreceptor cells, RST: recreational screen time, PRESSO: pleiotropy residual sum and outlier, SNP: single nucleotide polymorphism, S.E.: standard error, CI: confidence interval.

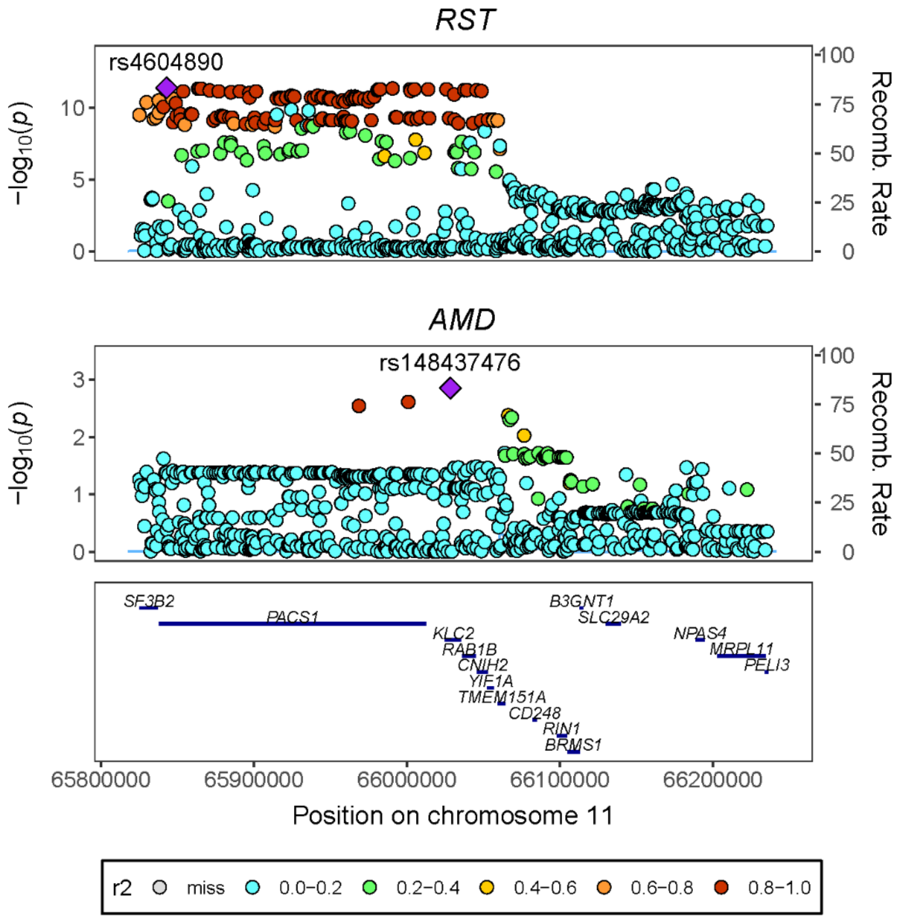

Figure S1 SNP regional association plot of RST and AMD in the KLC2 gene region

The left Y-axis shows the -log10 p-value of the genetic association of SNPs with the two traits. The right Y-axis shows the estimated recombination rates. The X-axis shows the genomic position. The top SNP is highlighted in purple. Surrounding SNPs within 200kb of the KLC2 gene are color-coded to reflect their linkage disequilibrium with the top SNP in r2.

RST: recreational screen time, SNP: single nucleotide polymorphisms, ST: screen time, AMD: age-related macular degeneration.

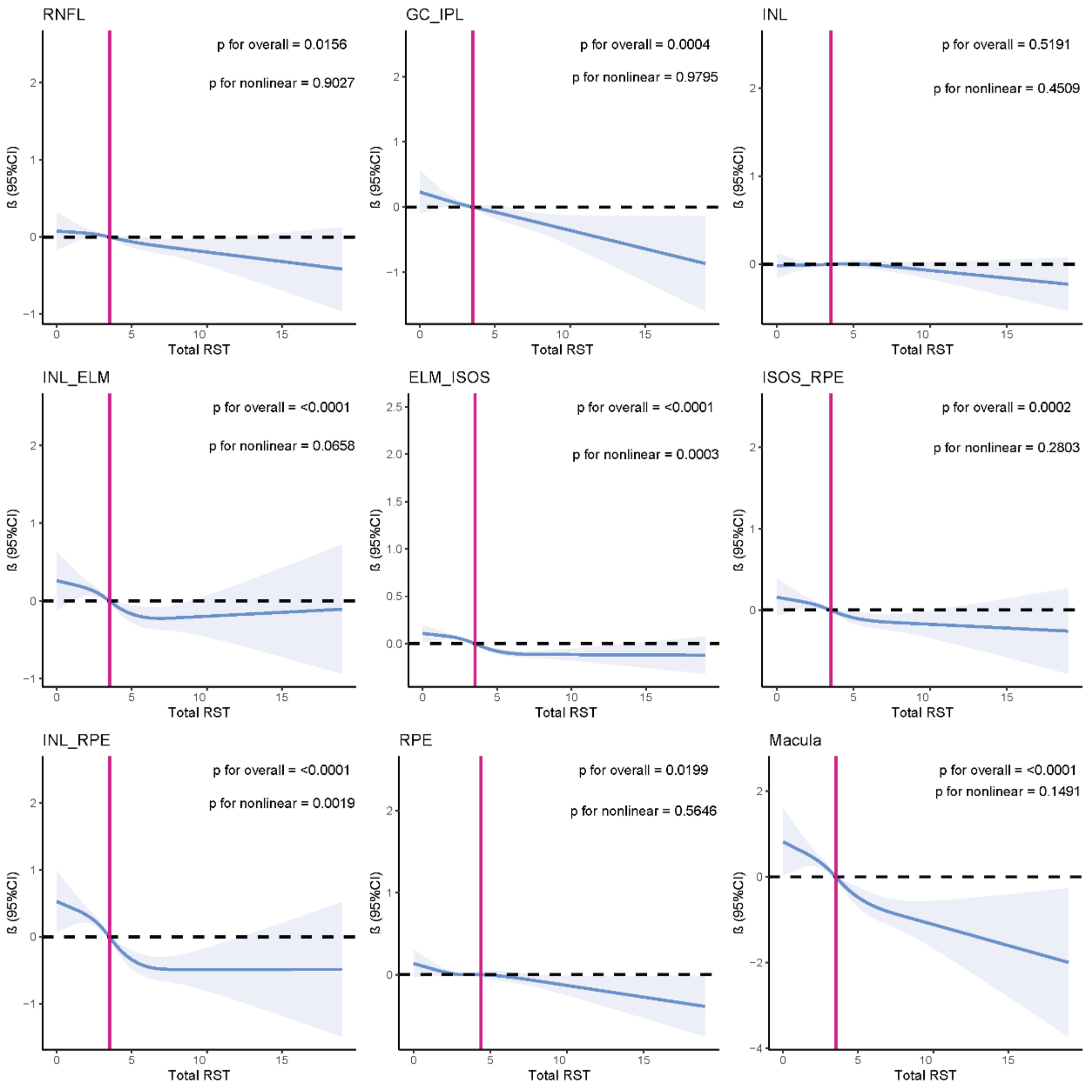

Figure S2 Association between total daily RST and retinal thickness evaluated by restricted cubic spline curves

The β (solid blue line) and 95%CI (light blue area) are from a multivariable adjusted linear regression model using restricted cubic spline curves with four knots (5th, 35th, 65th, and 95th percentiles). Adjustment includes age, sex, race, education, Townsend deprivation index, smoking status, history of hypertension, hyperlipidemia and cardiovascular diseases, visual acuity of the better eye, mean intraocular pressure and mean spherical equivalent of both eyes. The red line indicates the point where β = 0.

RST: recreational screen time, CI: confidence interval; RNFL: average retinal nerve fiber layer thickness across all subfields; GC-IPL: average ganglion cell-inner plexiform layer thickness across all subfields; INL: average inner nuclear layer thickness across all subfields; INL-ELM: average thickness measured between the inner nuclear layer to the external limiting membrane across all subfields, mainly reflecting photoreceptor cell axons, synaptic terminals, and nuclei; ELM-ISOS: average thickness measured between the external limiting membrane to the junction between the inner and outer photoreceptor segments across all subfields, mainly reflecting the inner segment of photoreceptor cells; ISOS-RPE: average thickness measured between the junction between the inner and outer photoreceptor segments to the retinal pigment epithelium across all subfields, mainly reflecting the outer segment of photoreceptor cells; INL-RPE: average thickness measured between the inner nuclear layer to the retinal pigment epithelium across all subfields, mainly reflecting the total photoreceptor layer thickness; RPE: average retinal pigment epithelium thickness across all subfields; Macula: average macular thickness measured from the inner limiting membrane to the retinal pigment epithelium across all subfield
